# Cereblon Control of Zebrafish Brain Size by Regulation of Neural Stem Cell Proliferation

**DOI:** 10.1016/j.isci.2019.04.007

**Published:** 2019-04-09

**Authors:** Hideki Ando, Tomomi Sato, Takumi Ito, Junichi Yamamoto, Satoshi Sakamoto, Nobuhiro Nitta, Tomoko Asatsuma-Okumura, Nobuyuki Shimizu, Ryota Mizushima, Ichio Aoki, Takeshi Imai, Yuki Yamaguchi, Arnold J. Berk, Hiroshi Handa

**Affiliations:** 1 Department of Nanoparticle Translational Research, Tokyo Medical University, 6-1-1, Shinjuku, Shinjuku-ku, Tokyo 160-8402, Japan; 2 PRESTO, JST, 4-1-8, Honcho, Kawaguchi, Saitama 332-0012 Japan; 3 National Institute of Radiological Sciences (NIRS), Chiba 263-8555, Japan; 4 School of Life Science and Technology, Tokyo Institute of Technology, Yokohama 226-8501, Japan; 5 National Center for Geriatrics and Gerontology (NCGG), Aichi 474-8511, Japan; 6 Department of Microbiology, Immunology, and Molecular Genetics, and Molecular Biology Institute, University of California, Los Angeles 90095, USA

**Keywords:** Cellular Neuroscience, Developmental Neuroscience, Molecular Neuroscience

## Abstract

Thalidomide is a teratogen that causes multiple malformations in the developing baby through its interaction with cereblon (CRBN), a substrate receptor subunit of the CRL4 E3 ubiquitin ligase complex. CRBN was originally reported as a gene associated with autosomal recessive non-syndromic mild mental retardation. However, the function of CRBN during brain development remains largely unknown. Here we demonstrate that CRBN promotes brain development by facilitating the proliferation of neural stem cells (NSCs). Knockdown of CRBN in zebrafish embryos impaired brain development and led to small brains, as did treatment with thalidomide. By contrast, overexpression of CRBN resulted in enlarged brains, leading to the expansion of NSC regions and increased cell proliferation in the early brain field and an expanded expression of brain region-specific genes and neural and glial marker genes. These results demonstrate that CRBN functions in the determination of brain size by regulating the proliferation of NSCs during development.

## Highlights

• CRBN is a determinant of head and brain size during zebrafish development
• Thalidomide causes a reduction in head and brain size by binding to CRBN
• CRBN prevents apoptosis and promotes NSC proliferation during brain development
• *crbn* overexpression results in a concomitant increase in neurons and glial cells

## Graphical Abstract

**Figure:**
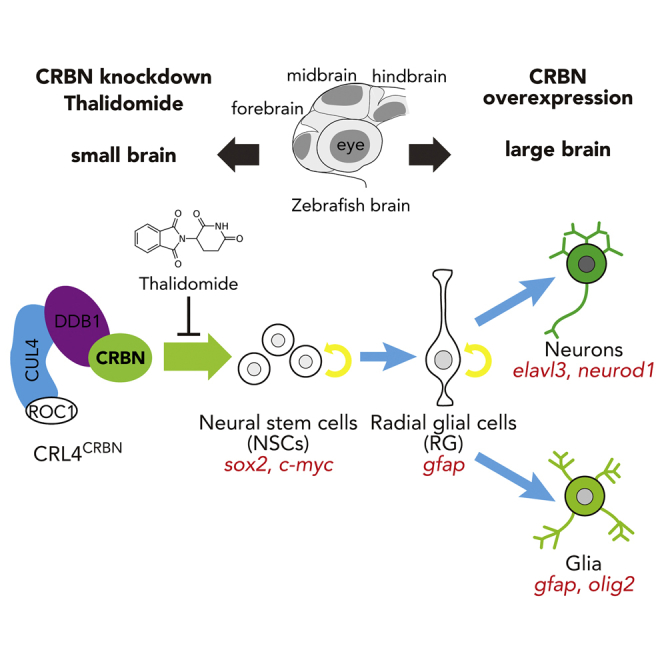

## Introduction

Brain size is primarily determined by the number of NSCs or precursor cells generated during development (Rakic, 2009, Lui et al., 2011; Florio and Huttner, 2014). Brain development is precisely controlled by the coordination between cell proliferation and differentiation of NSCs or precursor cells, which gives rise to correct expansion of the early precursor pool before the onset of differentiation (Ohnuma et al., 2001, Appolloni et al., 2008, Salomoni and Calegari, 2010). Although several genes have been reported as potent regulators of the expansion of undifferentiated precursor cells in the brain (Tiberi et al., 2012), the core determinant of the number of NSCs remains obscure.

Thalidomide is a well-known teratogen that causes multiple birth defects in limbs, ears, and eyes when administered during early pregnancy (Ito and Handa, 2012, Vargesson, 2015). Early prenatal exposure to thalidomide also causes autism (Miller et al., 2004), suggesting that brain development is also affected by thalidomide. Indeed, prenatal exposure to thalidomide causes microcephaly in rat embryos (Hallene et al., 2006, Fan et al., 2008). However, it remains ambiguous exactly how thalidomide has an impact on brain development.

Previously, we identified cereblon (CRBN) as a direct target of thalidomide and uncovered that CRBN is a subunit of the E3 ubiquitin ligase complex cullin-RING ligase 4 (CRL4) that is sensitive to thalidomide (Ito et al., 2010, Ito et al., 2011). The CRL4 E3 ubiquitin ligase CUL4–ROC1–DDB1–CRBN (CRL4^CRBN^) mediates the teratogenic and immunomodulatory effects of thalidomide and its derivatives lenalidomide and pomalidomide (Lopez-Girona et al., 2012, Matyskiela et al., 2016; see Figure 2A). DDB1, one of the subunits of CRL4^CRBN^, is required for cell proliferation through the p53-dependent pathway in the developing brain (Cang et al., 2006, Hu et al., 2015). Coincidentally, CRBN was originally reported as a gene responsible for autosomal recessive, non-syndromic mental retardation in humans (Higgins et al., 2004) and is highly expressed in the vertebrate brain (Higgins et al., 2010, Aizawa et al., 2011). In addition, it has been reported that CRBN also regulates the expression of functional large-conductance, Ca^2+^- and voltage-activated K^+^ (BK) channels, which are involved in neuronal excitability and epileptogenesis (Jo et al., 2005, Liu et al., 2014). Furthermore, forebrain-specific *Crbn* knockout mice show impairment in learning and memory (Rajadhyaksha et al., 2012). These lines of evidence suggest that CRBN is involved in normal brain functions at adult stages as well as during embryonic development. However, much remains to be elucidated regarding how CRBN functions in brain development.

Here, we present a function for the CRL4^CRBN^ E3 ubiquitin ligase complex in brain development. Knockdown of *crbn* gave rise to small brains in zebrafish embryos, as did thalidomide treatment or *cul4* depletion. Knockdown of *crbn* induced apoptosis through the p53-dependent pathway. Conversely, overexpression of *crbn* generated enlarged brains. In contrast to *crbn* knockdown phenotypes, we found that *crbn* overexpression caused an increase in the number of NSCs in the embryonic brain, resulting in the development of more neurons and glial cells. CRBN contributes to the determination of brain size through the regulation of Sox2 and c-Myc. These findings suggest a mechanism by which the E3 ubiquitin ligase activity of CRL4^CRBN^ regulates NSC proliferation during brain development.

## Results

### Treatment with Thalidomide Causes Small Brains in Zebrafish Embryos

**Figure 1:**
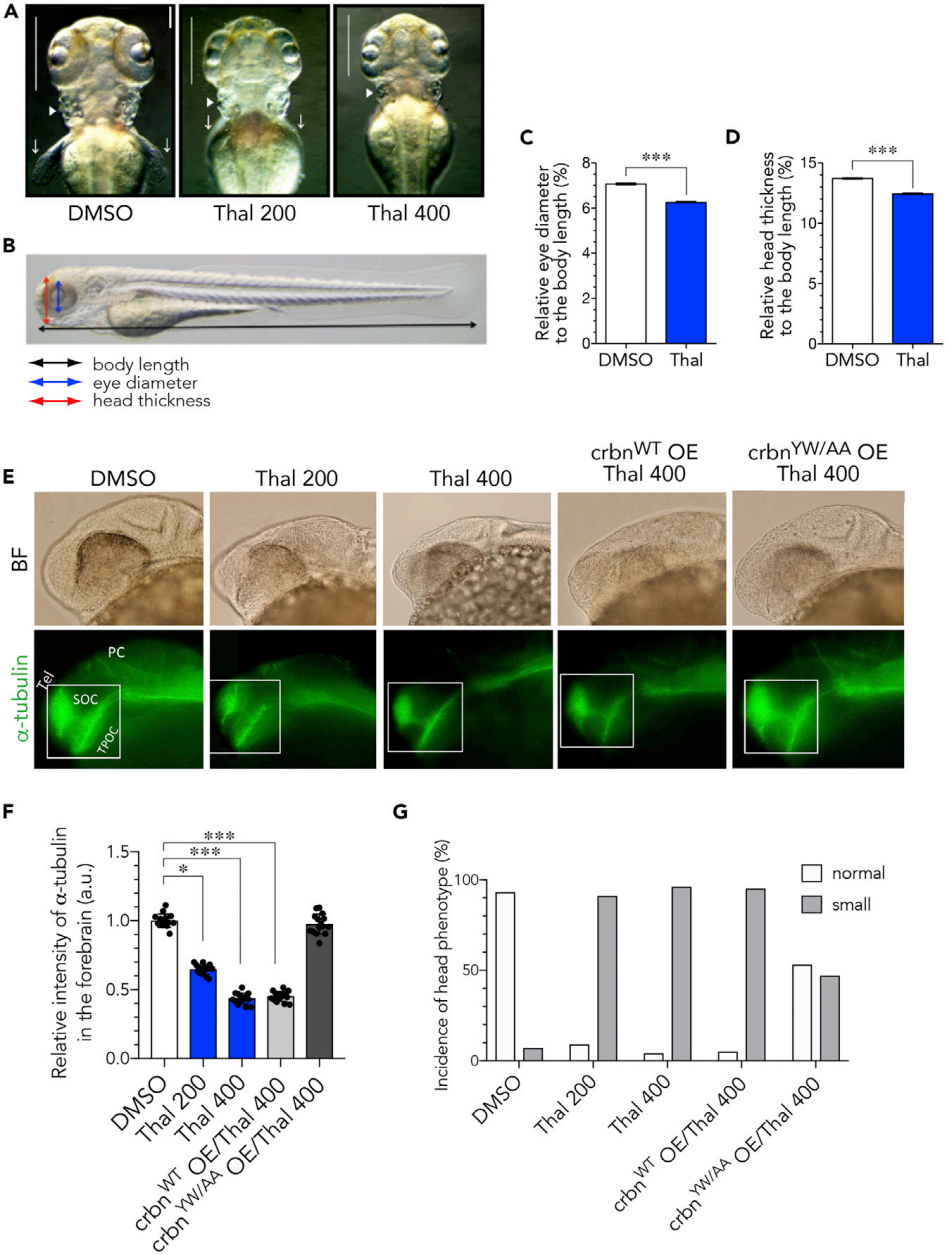
Thalidomide Retards Brain Development and Causes Microcephaly (A) Head morphology of 72-hpf zebrafish that were grown in the presence of 0.1% DMSO (left), 200 μM thalidomide (middle), or 400 μM thalidomide (right). Thin vertical lines indicate the distance between the rostral-most tip of olfactory bulbs and the temporal-most edge of eyes. Otic vesicles (arrowheads) and pectoral fins (arrows) are also indicated. (B) A schematic diagram depicting body length (black arrow), eye diameter (blue arrow) and head thickness (red arrow) that were used to determine ratios. (C and D) The ratios of eye diameter (blue arrow) in (C) and head thickness (red arrow) in (D) to body length (black arrow) of zebrafish that were grown with or without 400 µM thalidomide were determined and are shown as means ± SEM (n = 20 per group). (E) Primary neurons of 24-hpf embryos that were grown in the presence of 0.1% DMSO or 200 or 400 μM thalidomide. Where indicated, capped mRNA encoding *crbn*^*WT*^ or *crbn*^*YW/AA*^ was microinjected at the 1-cell stage before thalidomide treatment. Bright-field (BF) (upper panels) and fluorescence (lower panels) images of embryos immunostained with acetylated α-tubulin antibody are shown. Tel, telencephalon; SOC, supraoptic commissure; TPOC, tract of postoptic commissure; PC, posterior commissure. (F) Fluorescence intensity of acetylated α-tubulin-positive neural clusters in telencephalon and TPOC. Fluorescence intensities of the regions indicated with rectangles in (E) were measured and normalized to the intensity of DMSO-treated embryos and are shown as means ± SD (n = 15 per group). (G) Percent incidence of head phenotype (n = 100 per group in a single trial). The head sizes of 72-hpf embryos were classified based on the head-to-body ratio using the following criteria: ≥13%, normal; <13%, small. Scale bar, 50 μm. *p < 0.05, ***p < 0.001.

We reported earlier that thalidomide treatment of zebrafish embryos blocked the development of pectoral fins and otic vesicles (Ito et al., 2010). In addition to these effects, the head was smaller in thalidomide-treated fish than in untreated fish at 72 hours post fertilization (hpf) (Figure 1A). To measure the relative sizes of the head and eyes, we calculated the ratios of head size and eye diameter, respectively, to body length at 72 hpf (Figures 1B–1D). The average body length was not significantly different between untreated and thalidomide-treated fish (3.3 ± 0.07 mm in untreated fish, 3.2 ± 0.08 mm in fish treated with 400 μM thalidomide, n = 20 for each experiment). When fish were treated with 400 μM thalidomide, the average eye diameter was reduced to 89.9% ± 6.6% of that of untreated fish, the eye-body ratio being 6.9% ± 0.3% and 6.2% ± 0.3% in untreated and thalidomide-treated fish, respectively (Figure 1C). Similarly, thalidomide treatment reduced the average head size to 91.2% ± 4.0% of that of untreated fish, the head-body ratio being 13.5% ± 0.4% and 12.3% ± 0.5% in untreated and thalidomide-treated fish, respectively (Figure 1D).

Immunohistochemistry with antibody against acetylated α-tubulin showed that primary neurons were developmentally retarded in thalidomide-treated embryos. To gain insight into the concentration-dependent effects of thalidomide on brain development, we quantified the immunofluorescence intensity of acetylated α-tubulin antibody labeling major tracts of primary neurons such as telencephalic cluster, supraoptic commissure, and tract of postoptic commissure in the forebrain of control and thalidomide-treated embryos (Figure 1E, bottom, white open square). Primary neurons were developed normally in control embryos treated with 0.1% DMSO, whereas the intensity of acetylated α-tubulin staining in the forebrain was significantly decreased in embryos treated with 200 or 400 μM thalidomide (Figure 1F, n = 15). Of the control embryos 93% developed brains of normal size, whereas more than 90% of drug-treated embryos developed smaller brains (Figure 1G). These results suggest that thalidomide inhibits the development of primary neurons in zebrafish in a concentration-dependent manner.

### *crbn* Is Preferentially Expressed in the Brain of Zebrafish Embryos

We analyzed *crbn* expression by *in situ* hybridization of zebrafish embryos. Although weak expression was observed in whole embryos at earlier stages, *crbn* was broadly expressed at a high level in the head region at 30 hpf (Ito et al., 2010). Concordantly, *cul4a*, another component of CRL4^CRBN^, is also expressed in the head region at around the same stages at a high level (Ito et al., 2010). To confirm the specificity of *in situ* hybridization signals obtained, we performed *in situ* hybridization using sense and antisense probes. As a result, only faint background staining was obtained using the sense probe (Figure S1A).

At 56 hpf, *crbn* expression was confined to the cranial vasculature (Figure S1A, CV), retinal cells (open arrowheads in Figure S1A), and radial glial cells in the ventricular region (Figure S1A, RG, square brackets). In addition to the head region, *crbn* expression was detected in the trunk notochord (Figure S1A, nc). For comparison, expression of *fli1a*, a marker gene for head blood vessels, was visualized (Figure S1B). Expression patterns of *crbn* and *fli1a* were partially overlapping with each other, supporting the idea that *crbn* is expressed in the cranial vasculature. However, *crbn* signals were clearly detected in the ventricular region (Figure S1A, square brackets), on the dorsal side of the *fli1a*-expressing region (Figure S1A, white arrowheads). Thus their expression patterns are distinct in some respects. These results suggest the possibility that *crbn* is involved in the development of the central nervous system.

### Knockdown of *crbn* Causes Development of Small Brains and p53-Dependent Apoptosis

**Figure 2:**
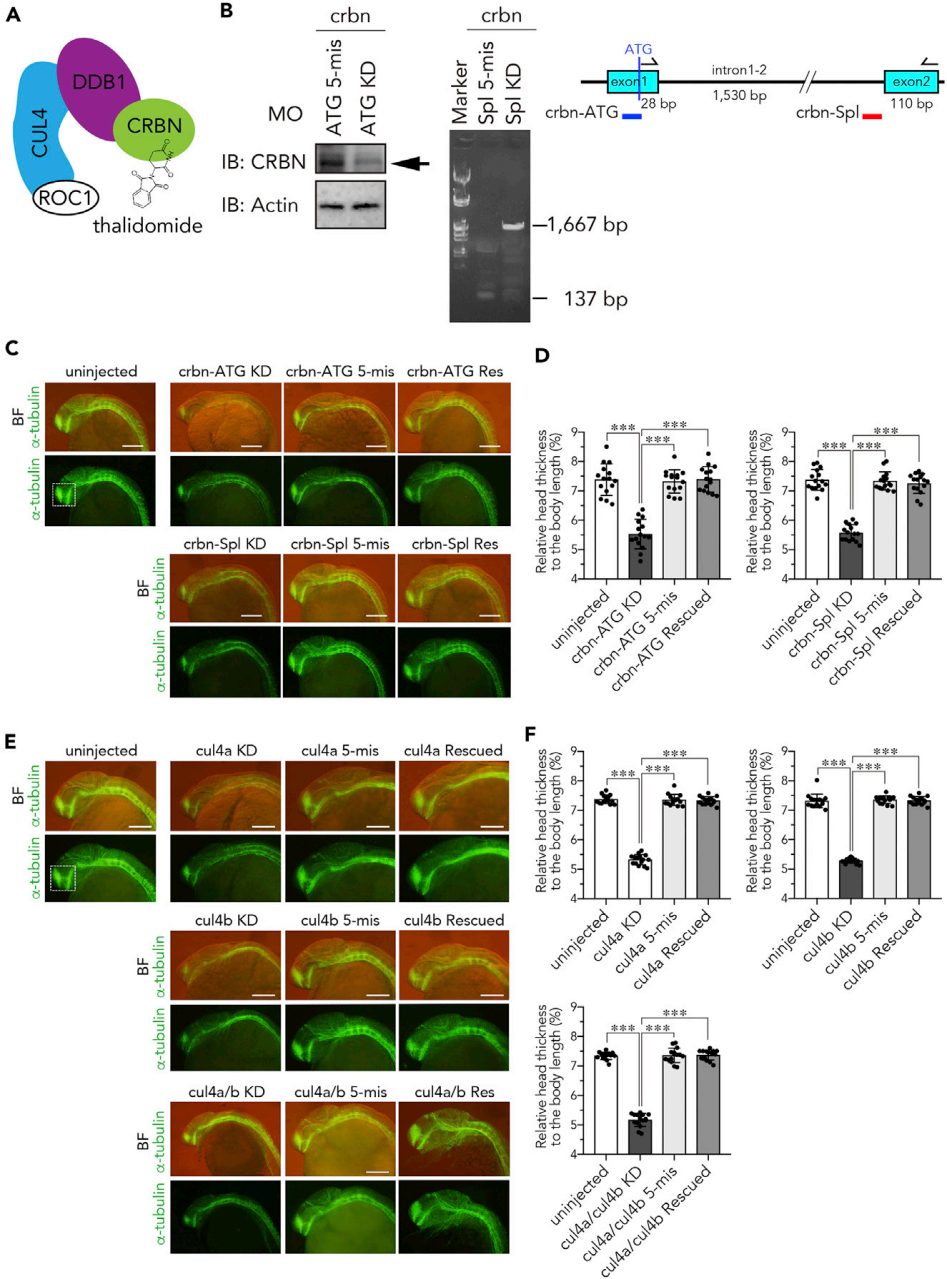
Knockdown of *crbn* or *cul4* Impairs Head Development (A) Schematic structure of CRL4^CRBN^ binding to thalidomide. (B) Immunoblot analysis (left) and RT-PCR analysis (right) of 24-hpf embryos injected with the indicated MOs against *crbn*. The indicated antibodies were used to analyze embryos injected with the translation-blocking MO. RT-PCR was performed using the indicated primer set to analyze embryos injected with the splice-blocking MO. The target site of the MO is indicated with red bar in the diagram. (C and E) One-cell-stage embryos were left uninjected or injected with the indicated MOs against *crbn* (C) or *cul4* (E) with or without corresponding mRNA and then subjected to immunostaining with anti-acetylated α-tubulin antibody at 24 hpf. Fluorescence images (lower panels) and those overlaid with bright-field images (upper panels) are shown. (D and F) The ratios of head thickness to body length of embryos that were left uninjected or injected with MOs against *crbn* (D) or *cul4* (F) are shown as means ± SD (n = 15 per group). Scale bar, 50 µm. ∗∗∗p < 0.001.

To examine the role of CRBN in brain development, we performed knockdown of *crbn* and two *cul4* variants (*cul4a* and *cul4b*) by microinjection of antisense morpholino oligonucleotides (MOs) that block translation or splicing. Because CRBN and CUL4 are subunits of the E3 ubiquitin ligase complex CRL4^CRBN^ that mediates teratogenic effects of thalidomide (Figure 2A), we expected that knockdown of these factors may phenocopy the effects of thalidomide, as was the case for pectoral fin malformation (Ito et al., 2010). Western blot analysis showed that CRBN expression was severely reduced by the translation-blocking MO against *crbn* (crbn-ATG) (Figure 2B). RT-PCR analysis also showed that the splice-blocking MO against *crbn* (crbn-Spl) inhibits the splicing of intron 1-2 (Figure 2B). In addition, reporter gene assay was employed to visualize the efficiency of translation inhibition in developing embryos. When a reporter gene containing the first exon of *crbn* and *egfp* was coinjected with the translation-blocking MO or the corresponding mismatch oligo, reporter gene expression in the head was significantly repressed by the coinjected MO (Figures S4A–S4C). Both crbn-ATG MO and crbn-Spl MO did not cause significant developmental delay (Figure S3A). Immunofluorescence staining with anti-acetylated α-tubulin antibody revealed that *crbn* knockdown embryos had smaller clusters of neurons in the forebrain including telencephalon and diencephalon and showed a significant decrease in fluorescence intensity in the forebrain (Figures 2C and S3C). In addition, the heads of *crbn* knockdown embryos were smaller than those of control embryos (Figure 2D). Similarly, knockdown of *cul4* variants significantly decreased the fluorescence intensity in the forebrain and the size of the head (Figures 2E and S3E). Single knockdown of *cul4a* or *cul4b* resulted in an approximately 30% reduction of fluorescence intensity, whereas double knockdown of *cul4a* and *cul4b* caused a more profound (approximately 50%) reduction, to a level comparable to that caused by *crbn* knockdown. The small brain phenotype induced by the knockdown of *crbn* or *cul4* was reversed by the coinjection of corresponding mRNA almost entirely (Figures 2D, 2F, and S3D), indicating that these factors are required for normal development of the brain.

To understand the underlying mechanism of the developmental defects, we examined whether apoptotic cells are increased in *crbn* knockdown embryos by immunohistochemistry using anti-activated Caspase-3 antibody. At 9 hpf, the late gastrulation stage, a large number of Caspase-3-positive cells were observed in a rostral-dorsal region including the brain anlagen in *crbn* knockdown embryos (Figure S4D, arrowheads). At 28 hpf, Caspase-3-positive cells were found throughout the brain and frequently detected as two clusters in the ventral diencephalon (Figure S4D, arrows). Apoptosis induced by *crbn* knockdown was significantly suppressed by simultaneous knockdown of *p53* (Figures S4E and S4F). Moreover, apoptosis induced by *crbn* knockdown was suppressed by the coinjection of *crbn* mRNA almost entirely (Figures S4G and S4H), indicating that *crbn* knockdown induces apoptosis at least in part through the *p53*-dependent pathway.

### Overexpression of *crbn* Causes Enlargement of the Head

**Figure 3:**
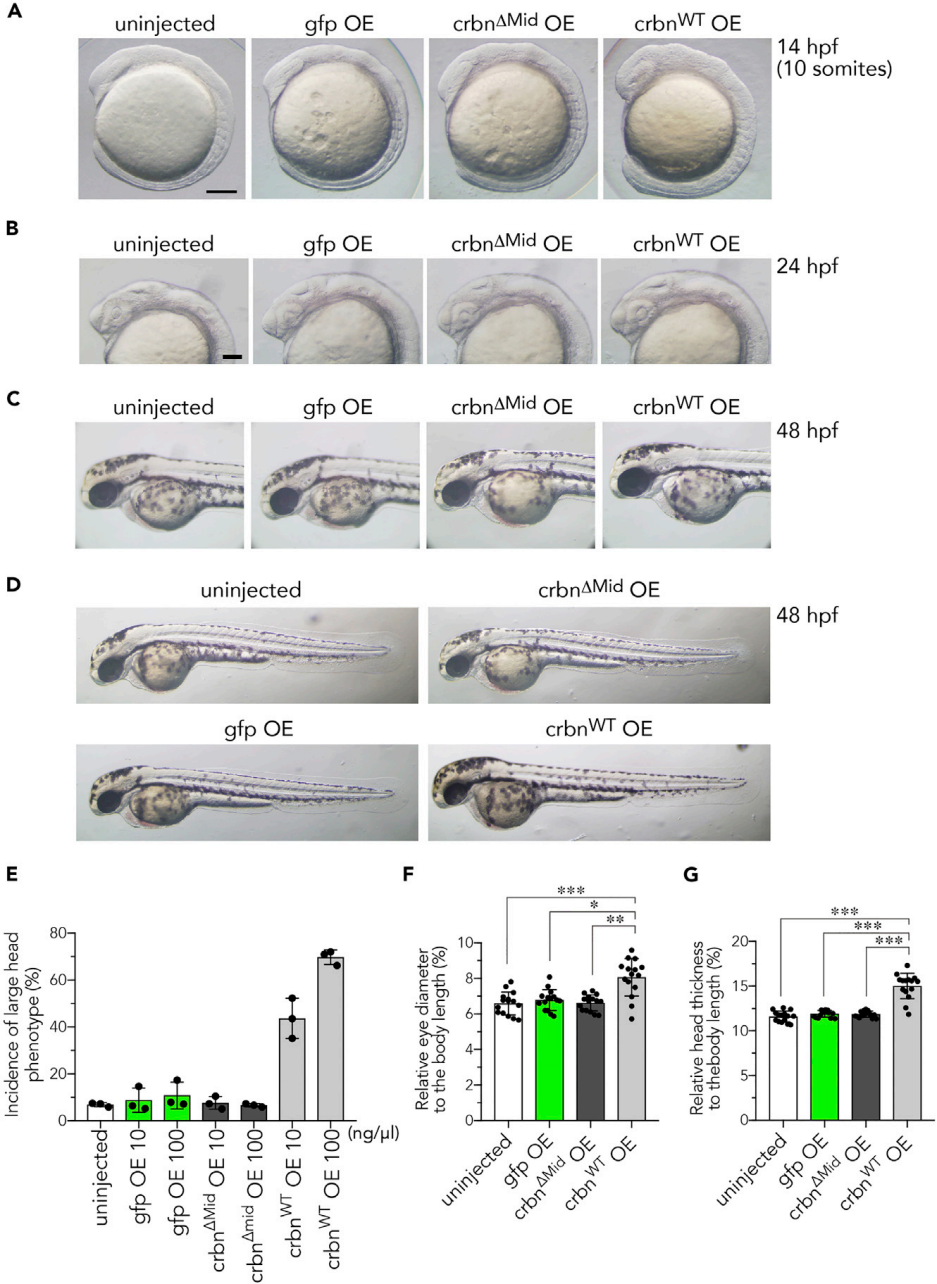
Overexpression of *crbn* Enlarges the Head (A–D) Head enlargement in *crbn*^*WT*^-overexpressing embryos at the 10-somite stage (14 hpf) (A), 24 hpf (B), and 48 hpf (C and D). One-cell-stage embryos were left uninjected or injected with approximately 300 pg of capped mRNA (300 ng/μL) encoding *gfp*, *crbn*^*ΔMid*^, or *crbn*^*WT*^. (E) One-cell-stage embryos were left uninjected or injected with capped mRNA at the indicated concentration. The head sizes of 14-hpf embryos were classified into “large” and “normal” based on the head-to-body ratio using the following criteria: ≤8%, normal; >8%, large. The fractions of large head embryos are shown as means ± SD (n = 3 per group). (F and G) The ratios of eye diameter (F) and head thickness (G) to body length were determined at 48 hpf as in Figures 1B–1D and are shown as means ± SD (n = 15 per group). Scale bar, 50 μm in (A and B). *p < 0.05, **p < 0.01, ***p < 0.001.

Next we examined the effects of overexpression of zebrafish *crbn* on brain development. One-cell stage embryos were left uninjected or injected with mRNA for *gfp*, wild-type *crbn* (*crbn*^*WT*^), or a functionally inactive mutant of *crbn* (*crbn*^*ΔMid*^). CRBN^ΔMid^ lacks a region required for DDB1 binding and therefore does not form a functional ubiquitin ligase complex (Ito et al., 2010). *crbn*^*WT*^ overexpression caused a remarkable expansion of the brain at 14 hpf (the 10-somite stage) and 24 hpf (Figures 3A and 3B). The optic vesicle was expanded, and thickness of the head was increased in *crbn*^*WT*^-overexpressing embryos, indicating that *crbn* has the ability to increase brain size during development (Figures 3A and 3B). At later stages (48 and 72 hpf), the head of *crbn*^*WT*^-overexpressing embryos was enlarged and heaved upward compared with control embryos (Figures 3C and S3C), whereas gross morphology including body axis appeared normal (Figures 3D and S3D). The effect of *crbn*^*WT*^ overexpression on head enlargement was dose dependent (Figure 3E). Furthermore, quantification of eye-to-body and head-to-body ratios revealed that both ratios were significantly increased in *crbn*^*WT*^-overexpressing embryos, but not in *gfp*- and *crbn*^*ΔMid*^-overexpressing embryos (Figures 3F and 3G, n = 15 for each experiment).

Next we asked whether *crbn* affects cell proliferation in the brain by immunostaining 24-hpf embryos with anti-phospho-histone H3 (pH3) antibody. In the telencephalon, *crbn* knockdown significantly reduced the number of pH3-positive proliferating cells, whereas *crbn* overexpression significantly increased the number of pH3-positive proliferating cells (Figure S6). These results suggest that *crbn* controls cell proliferation during brain development.

### Thalidomide-Induced Small Brain Size Is Mediated by Its Binding to CRBN

To confirm that thalidomide-induced retardation of brain development is caused by its binding to CRBN, we examined the effect of thalidomide on brain development in *crbn*^*YW/AA*^-overexpressing embryos. CRBN^YW/AA^ has two amino acid substitutions at the thalidomide-binding domain, Y374A and W376A, which together result in the loss of thalidomide-binding activity (Ito et al., 2010). In the absence of thalidomide, its overexpression resulted in enlargement of the head as *crbn*^*WT*^ overexpression did (Figures S3A and S3B). In the presence of thalidomide, overexpression of *crbn*^*YW/AA*^, but not of *crbn*^*WT*^, restored the immunofluorescence intensity of acetylated α-tubulin (Figures 1E and 1F) and the size of the head (Figures 1E and 1G). From these results, we concluded that CRBN mediates the effect of thalidomide on brain development, as is the case for its effect on limb development.

### CRBN Functions Downstream of the Six3-Lhx2b Pathway in the Determination of Brain Size

Previously, it was reported that Lhx2b mediates the activity of Six3 and together facilitates forebrain growth (Ando et al., 2005). The effects of knockdown or overexpression of *crbn* on brain development are quite similar to the phenotypes induced by knockdown or overexpression of the transcription regulators Six3 and Lhx2b in zebrafish (Ando et al., 2005, Ando and Okamoto, 2006). This similarity led us to study epistasis interactions between CRBN and the Six3-Lhx2b pathway by immunostaining neurons of 27-hpf embryos using anti-acetylated α-tubulin antibody. Acetylated α-tubulin-positive axons with normal projection patterns were increased in *crbn*^*WT*^-overexpressing embryos, suggesting an increase in the number of matured neurons (Figure S5A). Thus *crbn*^*WT*^-overexpressing embryos showed an excessive generation of neurons in the forebrain, as observed in *six3b*- or *lhx2b*-overexpressing embryos (Ando et al., 2005, Ando and Okamoto, 2006). We then asked whether developmental defects induced by the knockdown of *six3* or *lhx2b* are rescued by *crbn* overexpression. As reported previously (Ando et al., 2005), *six3* or *lhx2b* knockdown embryos showed a severe retardation of brain development with a significant decrease of acetylated α-tubulin intensity in the forebrain, and these defects were rescued by the coinjection of corresponding mRNA (Figures S5E, S5F, S5K, and S5L, n = 15). Note that the *six3* MO used in this study reportedly inhibits the expression of two functionally redundant genes, *six3a* and *six3b* (Ando et al., 2005); the small brain phenotype induced by this MO was reversed by the coinjection of *six3b* mRNA almost entirely. Remarkably, the defects induced by *six3* or *lhx2b* knockdown were restored by *crbn* overexpression (Figures S5B, S5C, S5H, and S5I, n = 15). By contrast, *six3b* overexpression did not rescue the phenotypes induced by *crbn* knockdown (Figures S5C and S5I, n = 15). Thus *crbn* compensated for the loss of *six3* or *lhx2b*, but not vice versa, indicating that CRBN functions downstream of the Six3-Lhx2b pathway in brain development.

### Overexpression of *crbn* Increases the Expression of Pluripotency Genes and the Number of NSCs in the Developing Brain

**Figure 4:**
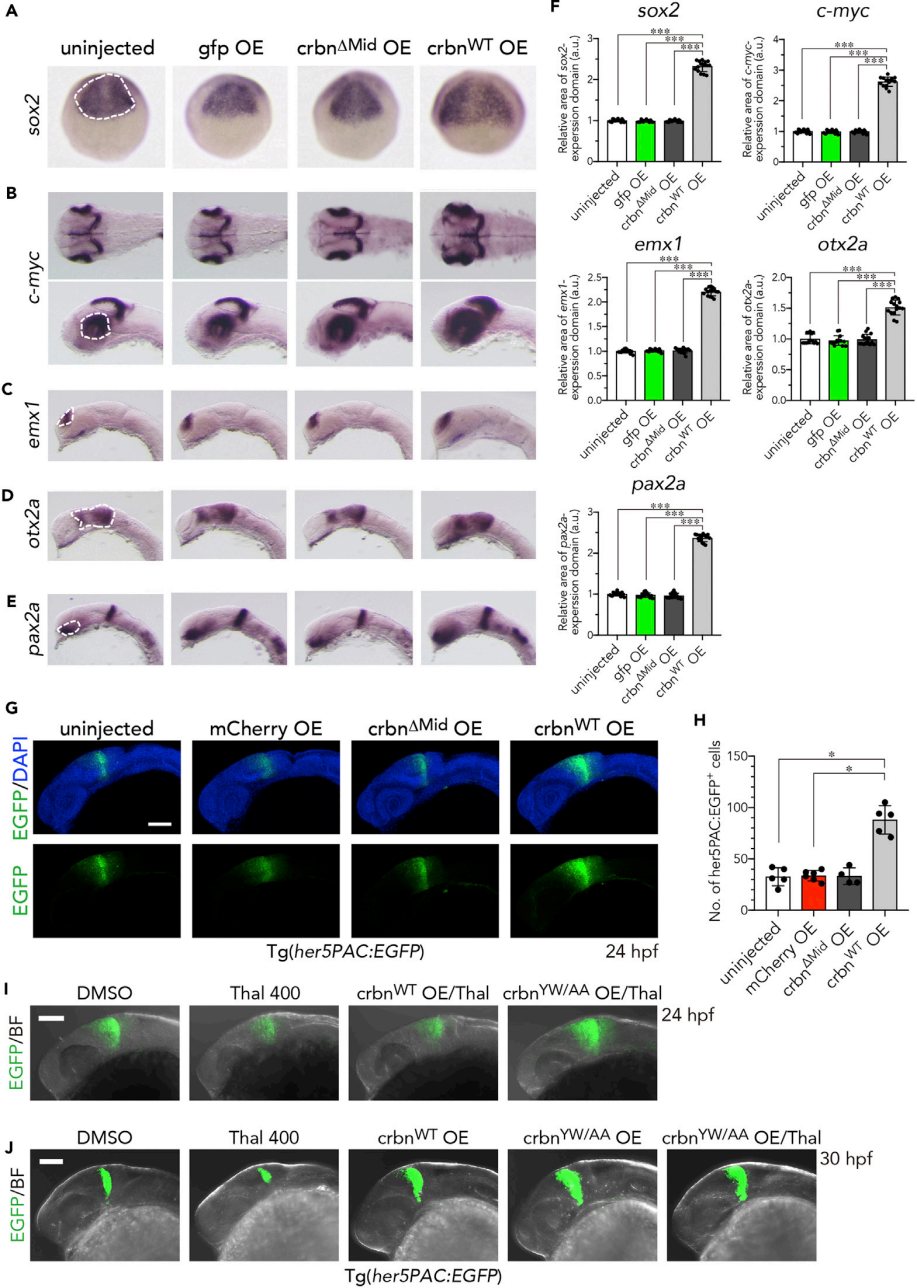
Increased Expression of Brain Marker Genes in *crbn*-overexpressing Embryos (A–E) *In situ* hybridization analysis for various brain marker genes in uninjected embryos or those overexpressing *gfp*, *crbn*^*ΔMid*^, or *crbn*^*WT*^. (A) *sox2* expression in the anterior brain field at 9 hpf. Dorsal view, anterior to the top. (B) *c-myc* expression at 30 hpf in the tectal proliferating zone and in the ciliary marginal zone in retina. Lateral view, anterior to the left. (C) *emx1* expression at 20 hpf in dorsal telencephalon. Lateral view, anterior to the left. (D) *otx2a* expression at 20 hpf in diencephalon. Lateral view, anterior to the left. (E) *pax2*.*1* expression at 20 hpf in optic stalk and MHB. Lateral view, anterior to the left. (F) The areas of expression domains for these genes, indicated with dashed lines in (A) to (E), were measured and normalized to the value of uninjected embryos and are shown as means ± SD. (n = 15 per group). (G) One-cell-stage embryos of *her5PAC*:*egfp* transgenic zebrafish were left uninjected or injected with capped mRNA encoding *mcherry*, *crbn*^*ΔMid*^, or *crbn*^*WT*^ and then analyzed at 24 hpf. Green fluorescence images (lower panels) and those overlaid with DAPI signals (upper panels) are shown. (H) The number of *her5PAC*:EGFP-positive cells were counted and are shown as means ± SD (n = 4–6 per group). (I and J) After mRNA microinjection, *her5PAC*:*egfp* transgenic embryos were grown in the presence of 0.1% DMSO or 400 μM thalidomide and analyzed at 24 hpf (I) or 30 hpf (J). Green fluorescence images overlaid with bright-field images are shown. Scale bar, 100 μm. *p < 0.05, ***p < 0.001.

To investigate the mechanism of CRBN-induced cell proliferation and head enlargement, we performed *in situ* hybridization to analyze the expression of developmental marker genes such as *sox2*, *c-myc*, *emx1*, *otx2a*, and *pax2a*. *sox2* and *c-myc* are known to induce pluripotency in both mouse and human somatic cells (Yamanaka and Blau, 2010), and *sox2* is an established marker for NSC self-renewal and pluripotency (Zappone et al., 2000). We found that *crbn*^WT^ overexpression increased and expanded the expression of these genes in the developing brain compared with uninjected embryos or those overexpressing *gfp* or *crbn*^*ΔMid*^ (Figures 4A–4E). *sox2* expression in the anterior brain region was spatially expanded at 9 hpf (Figure 4A), and *c-myc* expression in the tectal proliferating zone and the ciliary marginal zone of retina was increased and broadened at 30 hpf (Figure 4B). In addition, expression of the telencephalic marker *emx1* in the dorsal telencephalon was expanded in *crbn*^*WT*^-overexpressing embryos (Figure 4C). *otx2a* and *pax2a*, diencephalic markers that are expressed in the midbrain and in the optic stalk and midbrain-hindbrain boundary (MHB), respectively, also showed a broader expression in *crbn*^*WT*^-overexpressing embryos than in control embryos (Figures 4D and 4E). We measured the areas of expression domains for these genes (Figures 4A–4E, dashed lines) and found that all the areas were significantly increased in *crbn*^*WT*^-overexpressing embryos; approximately 230% for *sox2*, 260% for *c-myc*, 220% for *emx1*, 150% for *otx2a*, and 240% for *pax2a* compared with uninjected embryos (Figure 4F, n = 15). These results suggest that *crbn* overexpression activates the expression of genes required for neural development in the brain.

Next we used a transgenic line, Tg(*her5PAC*:*EGFP*), to visualize *her5*-positive NSCs in MHB (Tallafuss et al., 2003, Chapouton et al., 2006). *crbn*^*WT*^ overexpression expanded *her5*-positive regions in MHB and concomitantly increased the number of *her5*-positive NSCs at 24 hpf (Figures 4G and 4H). Treatment with 400 μM thalidomide markedly reduced *her5*-positive NSCs in uninjected embryos at 24 and 30 hpf (Figures 4I and 4J). Moreover, overexpression of *crbn*^*YW/AA*^, but not of *crbn*^*WT*^, reversed the effect of thalidomide (Figures 4I and 4J). These results indicate that thalidomide affects NSC development by binding to CRBN and affecting its ubiquitin ligase activity.

### The Number of Sox2-Positive NSCs Is Increased in the Brain Primordia of *Crbn*-Overexpressing Embryos from the Late Gastrulation Stage

**Figure 5:**
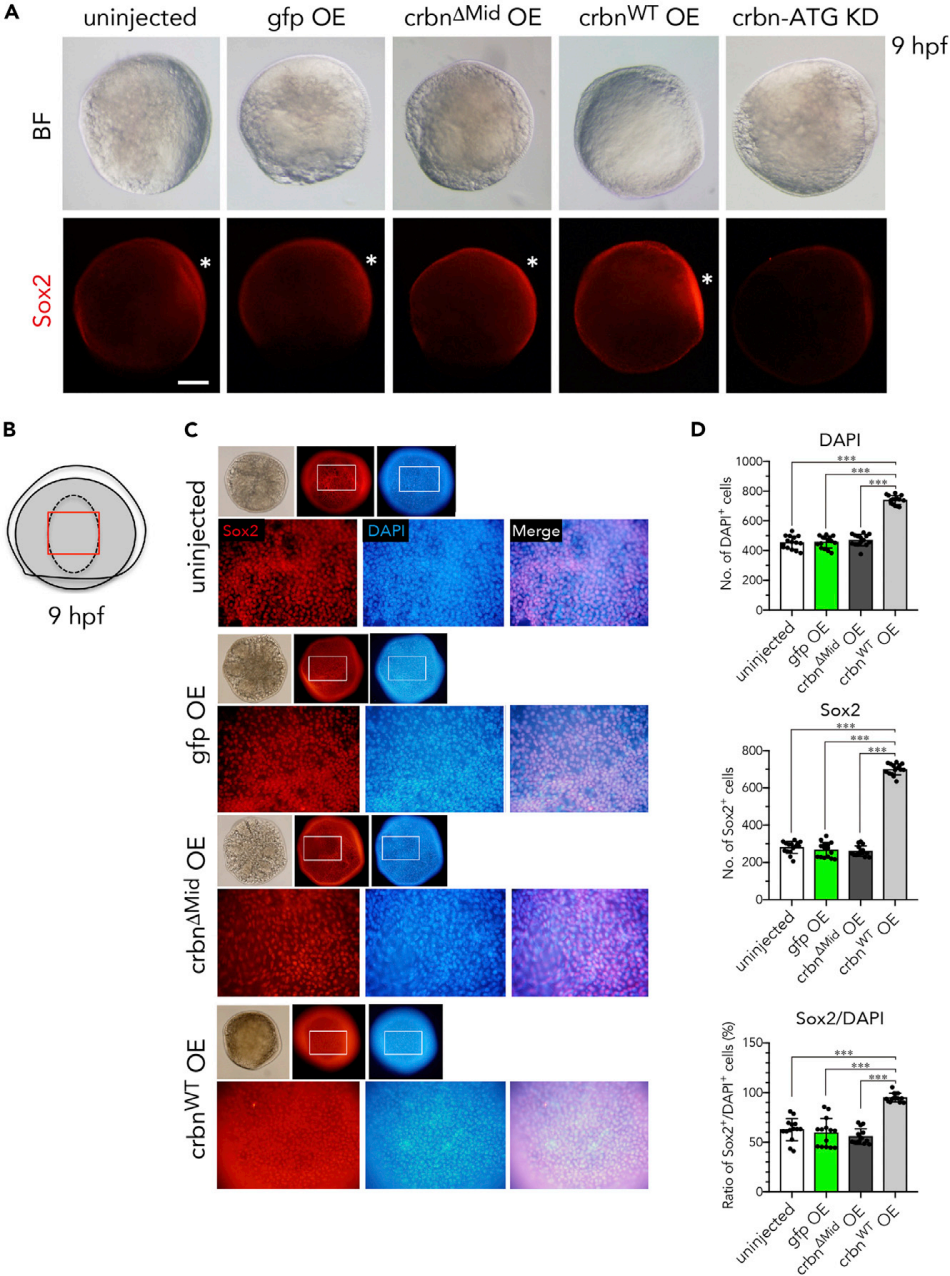
Sox2 Expression in *crbn*-Overexpressing Embryos (A) Embryos left uninjected or injected with mRNA encoding *gfp*, *crbn*^*ΔMid*^, or *crbn*^*WT*^ were immunostained using anti-Sox2 antibody and counterstained with DAPI at 9 hpf. Bright-field (upper panels) and fluorescence (lower panels) images are shown. Animal pole to the top, dorsal side to the right. Dorsal epidermis (the early brain field) is indicated with asterisks. (B) Illustration of 9-hpf embryos. Dorsal epidermis is indicated with dashed line. Lower panels in (C) correspond to the area indicated with red rectangle. (C) Close-up views of the early brain field stained with anti-Sox2 antibody and counterstained with DAPI. (D) Quantification and statistical analysis of the data shown in (C). DAPI-positive cells and Sox2-positive cells in the early brain field of 9-hpf embryos were counted and are shown as means ± SD (n = 15 per group). Scale bar, 150 μm. ***p < 0.001.

The pluripotency gene Sox2 is involved in the regulation of NSC and neural precursor cell proliferation in the developing brain from zebrafish to humans (Pevny and Placzek, 2005, Pevny and Nicolis, 2010). We studied the effect of *crbn* overexpression or knockdown on Sox2 expression more quantitatively at the protein level. At 9 hpf (the late gastrulation stage), *crbn*^*WT*^ overexpression increased the immunofluorescence signal of Sox2-positive cells in the dorsal region where NSCs and neural progenitor cells assemble as an anterior neural plate (Figure 5A, asterisks). By contrast, *crbn* knockdown dramatically decreased the immunofluorescence signal (Figure 5A). We counted the number of DAPI-positive cells and Sox2-positive cells in the anterior neural plate (Figure 5B, red rectangle). The number of DAPI-positive cells in the region of interest was not affected by the overexpression of *gfp* or *crbn*^*ΔMid*^, but was significantly increased ∼1.6-fold by the overexpression of *crbn*^*WT*^ (Figures 5C and 5D). The number of Sox2-positive cells was increased ∼2.6-fold by the overexpression of *crbn*^*WT*^, and as a result, the ratio of Sox2-positive cells to DAPI-positive cells was increased 1.5- to 1.6-fold by the overexpression of *crbn*^*WT*^ (Figures 5C and 5D), suggesting that *crbn*^*WT*^ overexpression increases the number of Sox2-positive NSCs in the brain primordia.

Similar analysis was performed at 24 hpf. In this case, fluorescence images were taken as a z series of optical sections at 50-μm intervals to accurately determine the number of DAPI-positive cells and Sox2-positive cells in a telencephalic region (indicated with rectangle in Figure S7A). As a result, essentially the same results were obtained (Figures S7B and S7C), indicating that the effect of *crbn*^*WT*^ overexpression on the number of NSCs is maintained from the late gastrulation stage to later stages of development.

### *crbn* Overexpression Leads to an Increase in Neurons and Glial Cells throughout the Enlarged Brain

**Figure 6:**
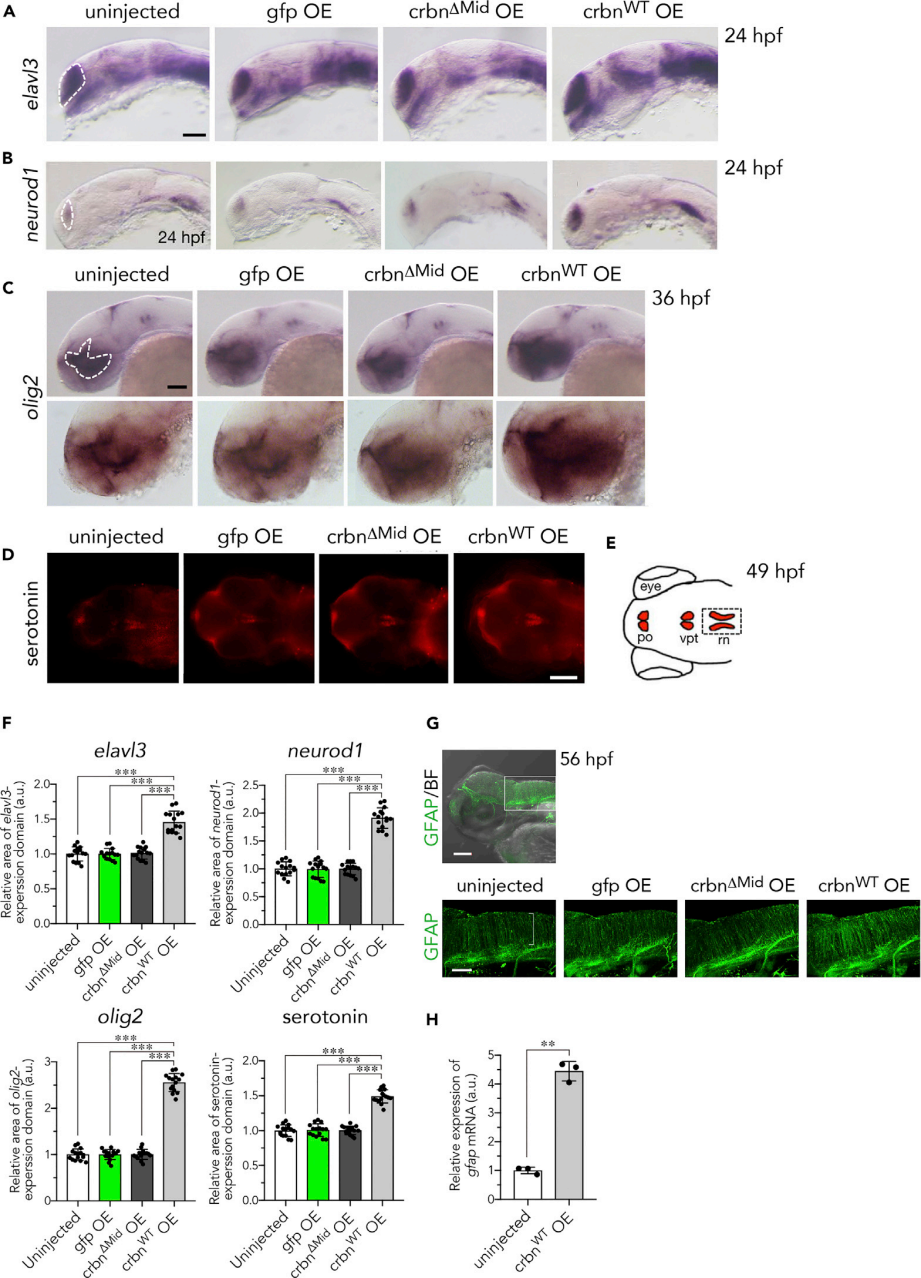
Increase of Neurons, Oligodendrocytes, GFAP-Positive Glia, and Radial Glia in *crbn*-Overexpressing Larvae (A–C) *In situ* hybridization analysis of uninjected embryos or those overexpressing *gfp*, *crbn*^*ΔMid*^, or *crbn*^*WT*^. (A) Expression of the neural marker *elavl3* (*huC*) in telencephalon at 24 hpf. (B) Expression of the neural and neural precursor marker *neurod1* in telencephalon and lateral longitudinal fascicles at 24 hpf. (C) Expression of the oligodendrocyte marker *olig2* in diencephalon at 36 hpf. Upper panels show lower-magnification images, and lower panels show higher-magnification images. (D) 49-hpf larvae immunostained with anti-serotonin antibody. (E) Schematic drawing of serotonin-positive cells at this stage. po, pineal organ; vpt, ventral posterior tubercle; rn, raphe nuclei. (F) The areas of expression domains for *elavl3*, *neurod1*, *olig2*, and serotonin, indicated with dashed lines in (A–C) and (E), were measured and normalized to the value of uninjected embryos and are shown as means ± SD (n = 15 per group). (G) Larvae at 56 hpf immunostained with antibody against the astrocyte marker GFAP. The area surrounded by rectangle in the upper panel is shown in lower panels. The fibers of radial glia are indicated by bracket. (H) Relative amounts of *gfap* mRNA in 11-hpf embryos were measured by quantitative RT-PCR and are shown as means ± SD (n = 3 per group). Scale bar, 100 μm in (D) and the upper panel in (G) and 50 μm in other panels. **p < 0.01, ***p < 0.001.

Next we studied potential consequences that the increase in NSCs could have on brain development. Expression of the post-mitotic neuronal marker *elavl3a* (*huc*) and the neural and neural progenitor marker *neurod1* were increased in *crbn*^*WT*^-overexpressing embryos (Figures 6A and 6B). In addition, expression of the oligodendrocyte marker *olig2* (Park et al., 2002) was increased in the forebrain of *crbn*^*WT*^-overexpressing embryos (Figure 6C). The areas of expression domains for these marker genes (Figures 6A–6C, dashed lines) were significantly increased in *crbn*^*WT*^-overexpressing embryos than in uninjected controls; approximately 146% for *elavl3a*, 191% for *neurod1*, and 256% for *olig2* compared with uninjected embryos (Figure 6F, n = 15).

*crbn* is reported to be expressed in serotonergic neurons in the raphe nuclei in mice (Aizawa et al., 2011). We therefore visualized serotonergic neurons in the diencephalon and hindbrain by immunostaining with anti-serotonin antibody and found that immunofluorescence signal in the raphe nuclei was significantly increased in *crbn*^*WT*^-overexpressing larvae at 49 hpf (Figures 6D and 6E, rn). The immunofluorescence signal in the raphe nuclei was increased approximately 1.5-fold by *crbn*^*WT*^ overexpression (Figure 6F, n = 15).

Next we examined the expression of glial fibrillary acidic protein (GFAP) by immunostaining with anti-GFAP monoclonal antibody, zrf-1. GFAP is a marker of radial glial cells and astrocytes, both of which are generated from NSCs (Trevarrow et al., 1990, Riley et al., 2004). Radial glial cells are known to function as a precursor of neurons and oligodendrocytes and as a scaffold to support neuronal migration (Kim et al., 2008). In 56-hpf *crbn*^*WT*^-overexpressing larvae, GFAP immunofluorescence signal was increased in radial glial or astrocyte fibers in the hindbrain (Figure 6G, bracket). A significant increase in *gfap* expression was also confirmed by quantitative RT-PCR using control and *crbn*^*WT*^-overexpressing embryos at 11 hpf (Figure 6H). These results suggest that *crbn* overexpression causes an increase in post-mitotic neurons, oligodendrocytes, serotonergic neurons, and radial glial cells or astrocytes in the brain.

## Discussion

Thalidomide causes different birth defects depending on exposure time during pregnancy in humans (Ito and Handa, 2012, Vargesson, 2015). Early prenatal exposure to thalidomide causes autism, suggesting a possibility that thalidomide affects brain development at early stages (Miller et al., 2004). Concordantly, prenatal exposure to thalidomide causes microcephaly in rat embryos (Hallene et al., 2006, Fan et al., 2008). However, it remains to be elucidated how thalidomide affects brain development in mammals. Previously, we showed that thalidomide exerts its teratogenic effect by binding to CRBN, a substrate receptor of the CRL4^CRBN^ E3 ubiquitin ligase complex (Ito et al., 2010). A number of subsequent studies have shown that binding of thalidomide or its derivatives affects CRL4^CRBN^ E3 ubiquitin ligase activity by altering its substrate specificity (Lopez-Girona et al., 2012, Matyskiela et al., 2016). However, the role of CRBN during brain development remains to be clarified. In this study, we used zebrafish as a vertebrate model for early brain development and demonstrated that thalidomide treatment of zebrafish embryos results in the development of small brains, suggesting that molecular mechanisms underlying thalidomide-induced microcephaly is conserved among vertebrates. We also showed that knockdown of *crbn* or *cul4* variants caused small brains in zebrafish embryos. These results suggest that CRL4^CRBN^ plays an essential role in brain development and in the regulation of brain size.

### CRBN Is Involved in the Survival of NSCs during Brain Development

We showed that knockdown of *crbn* induces apoptosis in the brain primordia from late gastrulation stages, leading to small brains in zebrafish embryos. Knockdown of another subunit of the CRL4^CRBN^ complex, *cul4a* or *cul4b*, also caused small brains. These results suggest that CRL4^CRBN^ is involved in the survival of NSCs in the brain at early developmental stages, thereby affecting the number of NSCs and later determining the size of the brain. We also showed that the apoptosis caused by *crbn* knockdown is mediated, at least in part, by p53. In mice, conditional inactivation of *ddb1*, another subunit of CRL4^CRBN^, in the brain and the lens leads to p53-dependent apoptosis in proliferating neuronal progenitor cells (Cang et al., 2006). Concordantly, a zebrafish mutation *ddb1*^m863^ shows enhanced apoptosis in the brain, most likely as a result of p53 activation, upregulation of *p21*^CIP1/WAF1^ (*cdkn1a*), and downregulation of cyclins *ccnd* and *ccna* in proliferating cells (Hu et al., 2015). Our results, taken together with these studies, corroborate the idea that CRL4^CRBN^ contributes to the survival of proliferating NSCs in the developing brain by inhibiting the p53-dependent apoptosis pathway.

### CRBN Promotes Cell Proliferation and Contributes to the Expansion of the Brain

We demonstrated that *crbn* overexpression facilitates cell proliferation and gives rise to more neurons and glial cells, leading to an enlarged brain. The enlargement appears to occur uniformly by enhancing the proliferation of neuroepithelial cells throughout the brain, including NSCs. In support, various developmental marker genes expressed in different brain regions were ubiquitously expanded, and GFP-positive NSCs in MHB were increased in *crbn*-overexpressing embryos. A plausible explanation for these observations is that an increase in the number of NSCs leads to the production of higher numbers of neurons and glial cells and eventually leads to an increase in brain size. Pluripotency genes, such as Sox2, play important roles in the regulation of NSC and precursor cell proliferation in the developing brain from zebrafish to humans (Pevny and Nicolis, 2010). Sox2 functions with c-Myc in the embryonic nervous system from the earliest stages of development (Archer et al., 2011, Pevny and Nicolis, 2010, Pevny and Placzek, 2005, Wegner and Stolt, 2005). In support, when *crbn* was overexpressed, Sox2 was highly induced in the brain primordia at the late gastrulation stage and in telencephalon at 24 hpf. Therefore Sox2-dependent survival and proliferation of NSCs in the presumptive brain region is a plausible target of CRL4^CRBN^ in the control of brain size.

### CRBN Is a Determinant of Brain Size during Development

Several genes associated with microcephaly and macrocephaly have been identified in humans (Kaindl et al., 2010, Williams et al., 2008). Growing evidence for the function of these genes illustrates that brain size is determined by the number of NSCs through the regulation of proliferation, cell cycle, cell survival, and apoptosis (Sun and Hevner, 2014). It is suggested that defects in mitotic spindle organization affect proper divisions of NSCs and lead to autosomal recessive primary microcephaly (Kaindl et al., 2010). On the other hand, expansion of NSCs generates macrocephaly, an abnormally large head caused by enlargement of the brain. The phosphatidylinositol 3-kinase/AKT3 signaling pathway and the tumor suppressor gene product PTEN play critical roles in controlling brain size and are implicated in macrocephaly (DiLiberti, 1998, Groszer et al., 2001, Poduri et al., 2012, Riviere et al., 2012, Song et al., 2012).

In this study, we demonstrated that the expression level of *crbn* determines brain size during development. Our findings suggest a molecular mechanism for controlling brain size by which CRL4^CRBN^ regulates ubiquitination and proteosomal degradation of inhibitor(s) of NSC proliferation. This study may open up the possibility for the use of thalidomide derivatives in controlling NSC proliferation for medical treatment.

### Limitations of the Study

According to recently published guidelines for MO use in zebrafish (Stainier et al., 2017), MOs should be used alongside mutant(s) for the corresponding gene. However, we have not directly compared he morphant and mutant phenotypes for the genes studied in this article. Therefore, we cannot rule out the possibility that off-target effects were misinterpreted as specific effects, although we checked the specificity of morphant phenotypes by performing rescue experiments for most of the MOs used in this study.

## Methods

All methods can be found in the accompanying Transparent Methods supplemental file.

## Supplemental Information

Document S1. Transparent Methods and Figures S1–S7
