# Supplementary material for: Cereblon Control of Zebrafish Brain Size by Regulation of Neural Stem Cell Proliferation
Source: iScience. 2019 Apr 9;15:95–108. doi: 10.1016/j.isci.2019.04.007 (PMC6501120; doi:10.1016/j.isci.2019.04.007)
Supplement: Document S1. Transparent Methods and Figures S1–S7 [file mmc1.pdf]

ISCI, Volume *15*

Supplemental Information

Cereblon Control of Zebrafish Brain Size by Regulation of Neural Stem Cell Proliferation

Hideki Ando, Tomomi Sato, Takumi Ito, Junichi Yamamoto, Satoshi Sakamoto, Nobuhiro Nitta, Tomoko Asatsuma-Okumura, Nobuyuki Shimizu, Ryota Mizushima, Ichio Aoki, Takeshi Imai, Yuki Yamaguchi, Arnold J. Berk, and Hiroshi Handa

Figure S1

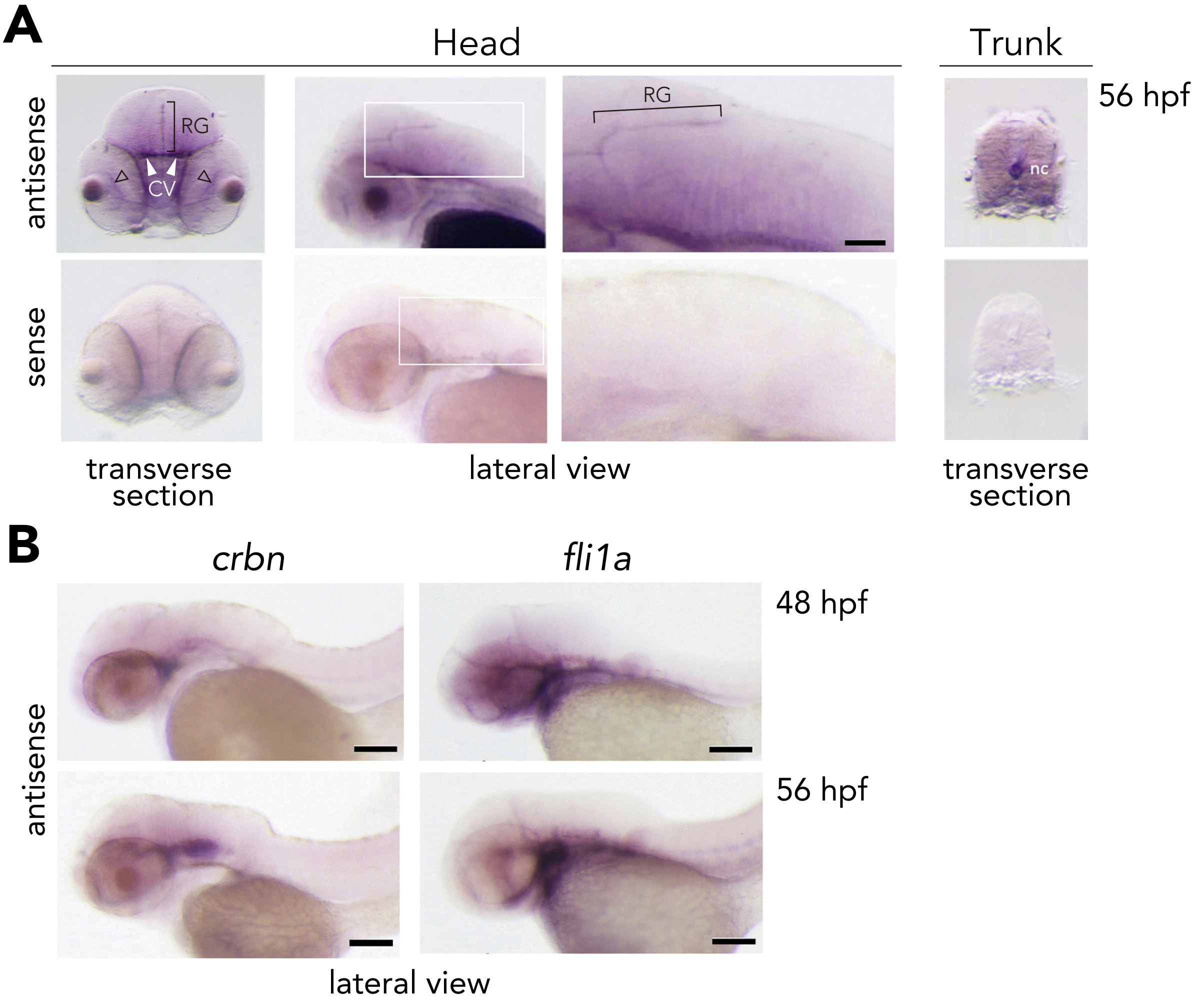
A
Head
Trunk
56 hpf
antisense
RG
CV
RG
nc
sense
transverse section
lateral view
transverse section
B
crbn
fli1a
48 hpf
antisense
56 hpf
lateral view

Figure S1. Expression Pattern of *crbn* mRNA in the Head of Zebrafish Embryos, Related to Figure 1.

(A) *In situ* hybridization was carried out to visualize the expression pattern of *crbn* at 56 hpf. To check background staining, antisense (upper panels) and sense (lower panels) probes was used. From left to right, cross sections of the head, lateral views, close-up lateral views of the regions indicated with rectangles in the previous panels, cross sections of the trunk. The shapes of the cross sections look different because one of them was deformed by the pressure from a cover glass. White arrowheads denote cranial vasculature (CV). Open arrowheads denote retinal cells. Square brackets denote radial glial cells (RG). nc, notochord. (B) Expression patterns of *crbn* and *fli1a* were compared at 48 hpf and 56 hpf. Scale bar, 50 μm.

Figure S2

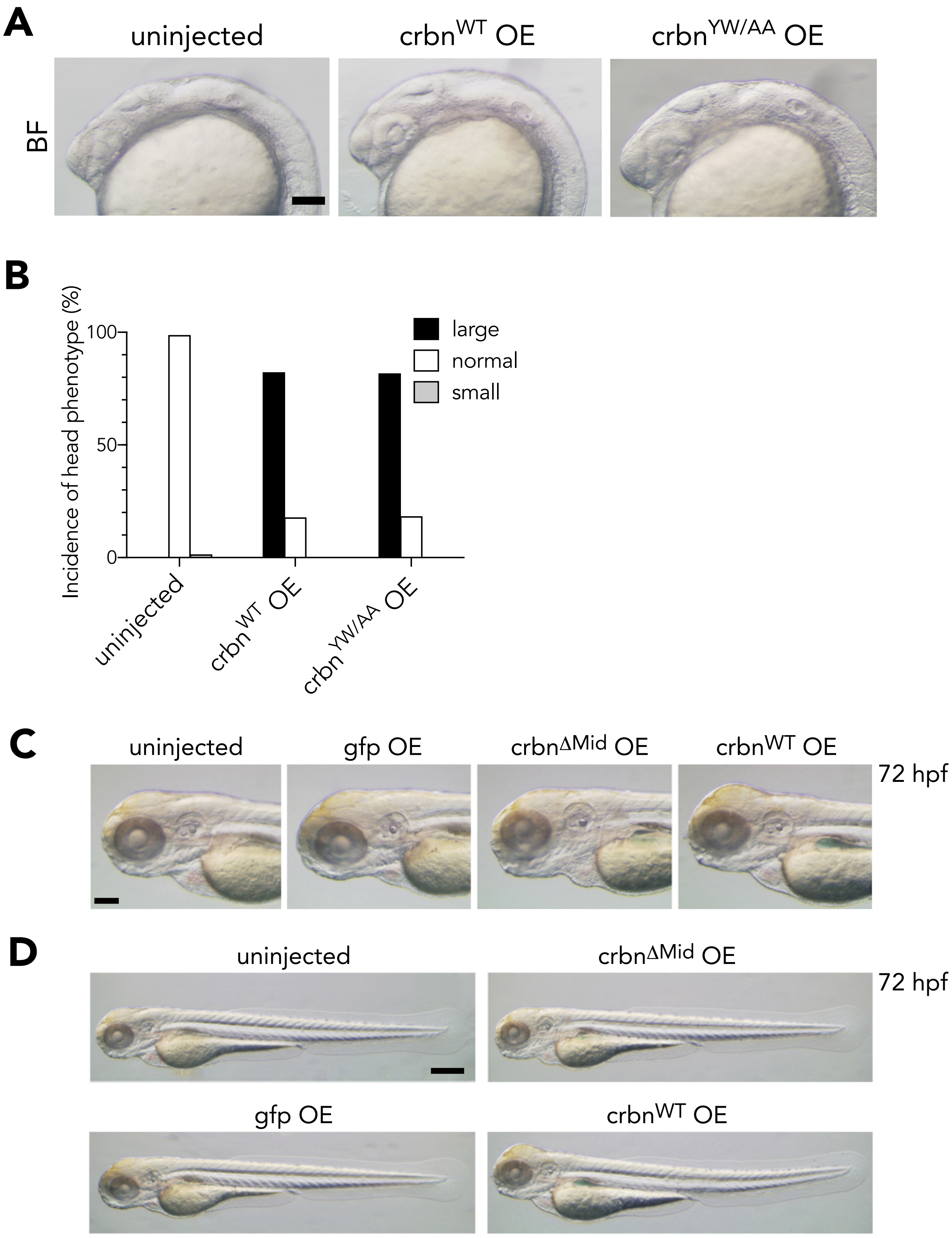
A
uninjected
crbnWT OE
crbnYW/AA OE
BF
B
Incidence of head phenotype (%)
100
50
0
large
normal
small
uninjected
crbn WT OE
crbn YW/AA OE
C
uninjected
gfp OE
crbnΔMid OE
crbnWT OE
72 hpf
D
uninjected
crbnΔMid OE
72 hpf
gfp OE
crbnWT OE

Figure S2. Morphology and Incidence of *crbn*^{WT}- and *crbn*$^{YW/AA}$-overexpressing Embryos, Related to Figures 1 and 3.

(A) Morphology of 24-hpf embryos that were left uninjected or injected with mRNA encoding *crbn*^{WT} or *crbn*$^{YW/AA}$. (B) Percent incidence of large head phenotype. The head sizes of 24-hpf embryos were classified into "large," "normal," and "small" based on the head-to-body ratio, and their fractions are shown. n = 77 (uninjected), 129 (*crbn*^{WT}), and 120 (*crbn*$^{YW/AA}$). Scale bar, 50 µm. (C and D) Head enlargement in *crbn*^{WT}-overexpressing embryos at 72 hpf. One-cell stage embryos were left uninjected or injected with approximately 300 pg of capped mRNA (300 ng/µl) encoding *gfp*, *crbn*$^{\Delta Mid}$, or *crbn*^{WT}.

Figure S3

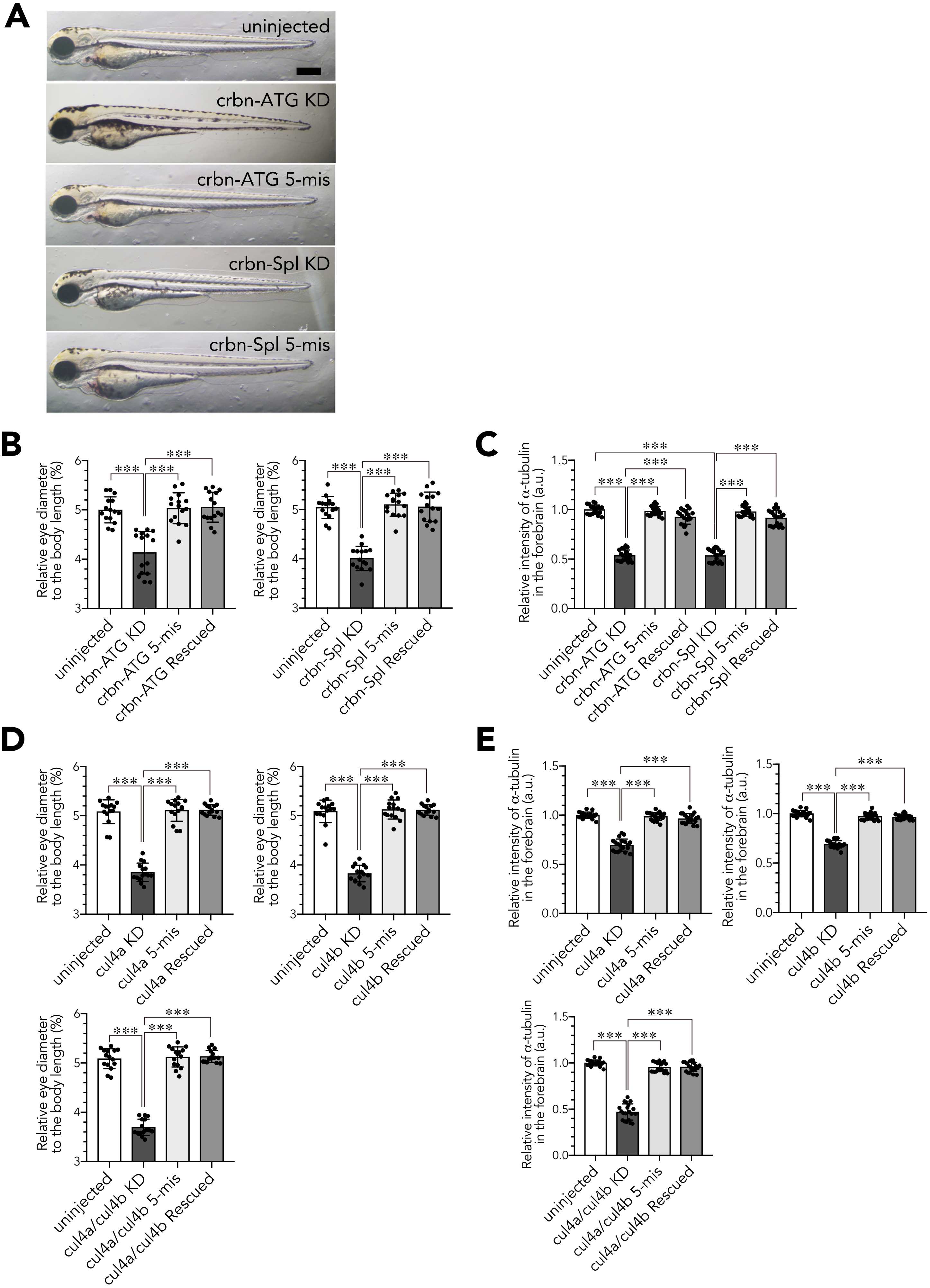

A
uninjected
crbn-ATG KD
crbn-ATG 5-mis
crbn-Spl KD
crbn-Spl 5-mis
B
Relative eye diameter to the body length (%)
***
uninjected
crbn-ATG KD
crbn-ATG 5-mis
crbn-ATG Rescued
crbn-Spl KD
crbn-Spl 5-mis
crbn-Spl Rescued
C
Relative intensity of α-tubulin in the forebrain (a.u.)
D
cul4a KD
cul4a 5-mis
cul4a Rescued
cul4b KD
cul4b 5-mis
cul4b Rescued
cul4a/cul4b KD
cul4a/cul4b 5-mis
cul4a/cul4b Rescued
E

Figure S3. Knockdown of *crbn* or *cul4* Impairs Brain Development, Related to Figure 2.

(A) Gross morphology of 72-hpf larvae uninjected or injected with the indicated MOs. (B and D) The ratios of eye diameter to body length of embryos that were left uninjected or injected with MOs against *crbn* (B) or *cul4* (D) are shown as means ± SD (n = 15 per group). (C and E) Quantification and statistical analysis of the data shown in Figures 2C and 2E. Fluorescence intensities of the regions indicated with rectangles in Figures 2C and 2E were measured and normalized to the intensities of uninjected embryos and are shown as means ± SD (n = 20 per group). Scale bar, 50 μm. ***, $P < 0.001$.

Figure S4

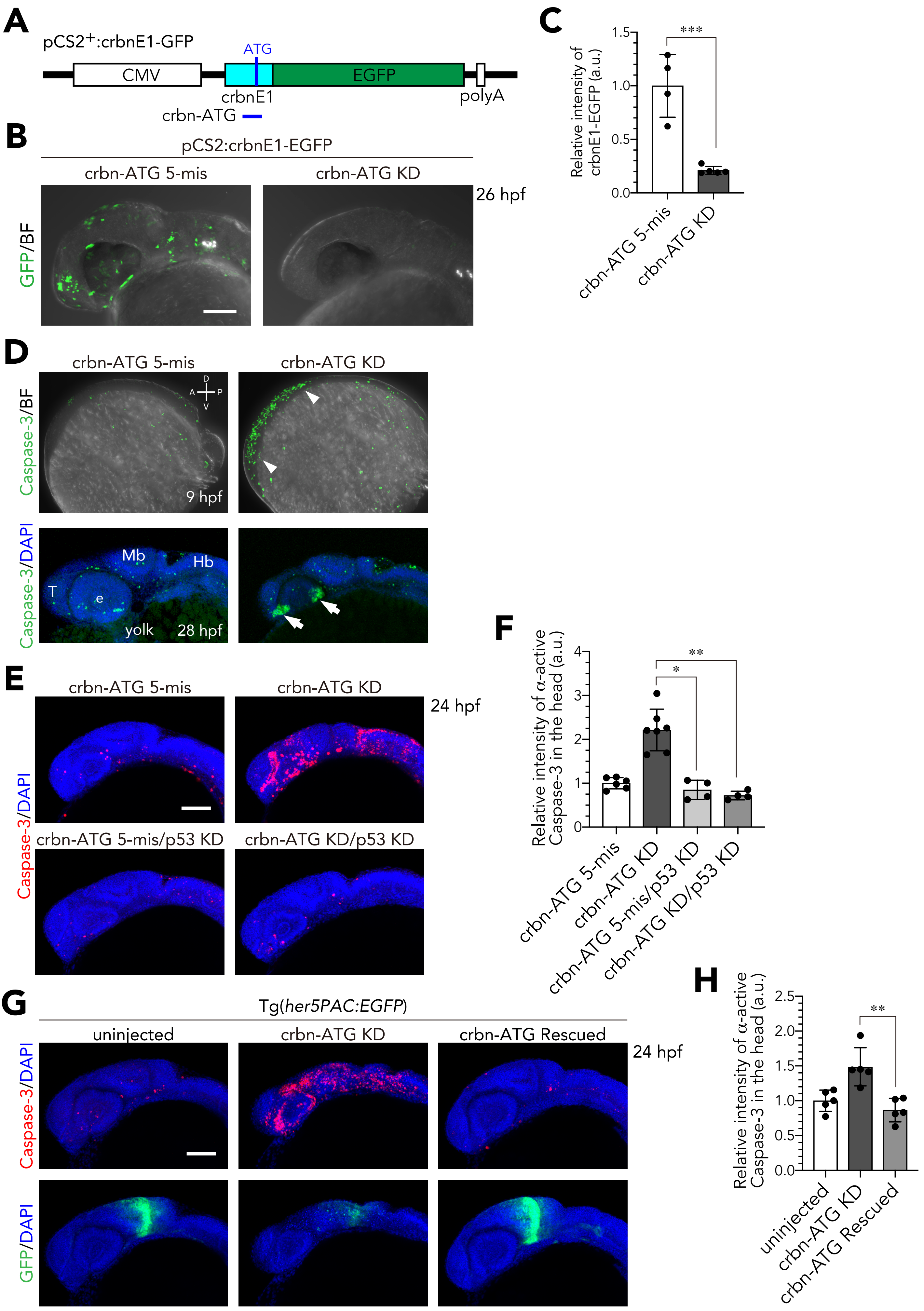

A
pCS2+:crbnE1-GFP
ATG
CMV
EGFP
crbnE1
polyA
crbn-ATG
B
pCS2:crbnE1-EGFP
crbn-ATG 5-mis
crbn-ATG KD
26 hpf
GFP/BF
C
Relative intensity of crbnE1-EGFP (a.u.)
***
1.5
1.0
0.5
0.0
crbn-ATG 5-mis
crbn-ATG KD
D
crbn-ATG 5-mis
crbn-ATG KD
Caspase-3/BF
D
A
P
V
9 hpf
Caspase-3/DAPI
Mb
Hb
T
e
yolk
28 hpf
E
crbn-ATG 5-mis
crbn-ATG KD
24 hpf
Caspase-3/DAPI
crbn-ATG 5-mis/p53 KD
crbn-ATG KD/p53 KD
F
Relative intensity of α-active Caspase-3 in the head (a.u.)
**
*
4
3
2
1
0
crbn-ATG 5-mis
crbn-ATG KD
crbn-ATG 5-mis/p53 KD
crbn-ATG KD/p53 KD
G
Tg(her5PAC:EGFP)
uninjected
crbn-ATG KD
crbn-ATG Rescued
24 hpf
Caspase-3/DAPI
GFP/DAPI
H
Relative intensity of α-active Caspase-3 in the head (a.u.)
**
2.5
2.0
1.5
1.0
0.5
0.0
uninjected
crbn-ATG KD
crbn-ATG Rescued

Figure S4. Knockdown of *crbn* Induces p53-dependent Apoptosis in the Brain, Related to Figure 2.

(A) Schematic structure of pCS2^{+}:*crbn*E1-EGFP. Exon 1 from *crbn* (crbnE1) containing the initiation codon was fused to *egfp* in frame. (B) pCS2^{+}:*crbn*E1-EGFP was coinjected with the indicated MO, and green fluorescence in the head was visualized at 26 hpf. (C) Fluorescence intensities of EGFP in the trunk region were determined and normalized to the intensities of control embryos and are shown as means ± SD (n = 5 per group). (D) Control and *crbn*-knockdown embryos were immunostained with anti-active Caspase-3 antibody and counterstained with DAPI at 9 hpf (top) and 28 hpf (bottom). Arrowheads indicate the prospective head region. Arrows indicate clusters of apoptotic cells frequently observed in ventral diencephalon. (E) Following microinjection of *crbn* and/or *p53* MOs, 24-hpf embryos were immunostained with anti-active Caspase-3 antibody and counterstained with DAPI. e, eye; Hb, hindbrain; Mb, midbrain; T, telencephalon. (F) Fluorescence intensities of anti-active Caspase-3 antibody in the head were determined and normalized to the intensities of control embryos and are shown as means ± SD (n = 4 to 7 per group). (G) *crbn* knockdown and rescue experiments were performed using transgenic zebrafish expressing *her5PAC*:EGFP. Then, 24-hpf embryos were immunostained with anti-active Caspase-3 antibody and counterstained with DAPI. (H) Fluorescence intensities of anti-active Caspase-3 antibody in the head were determined and normalized to the intensities of control embryos and are shown as means ± SD (n = 5 per group). Scale bar, 100 µm. *$P < 0.05$, **$P < 0.01$, ***, $P < 0.001$.

Figure S5

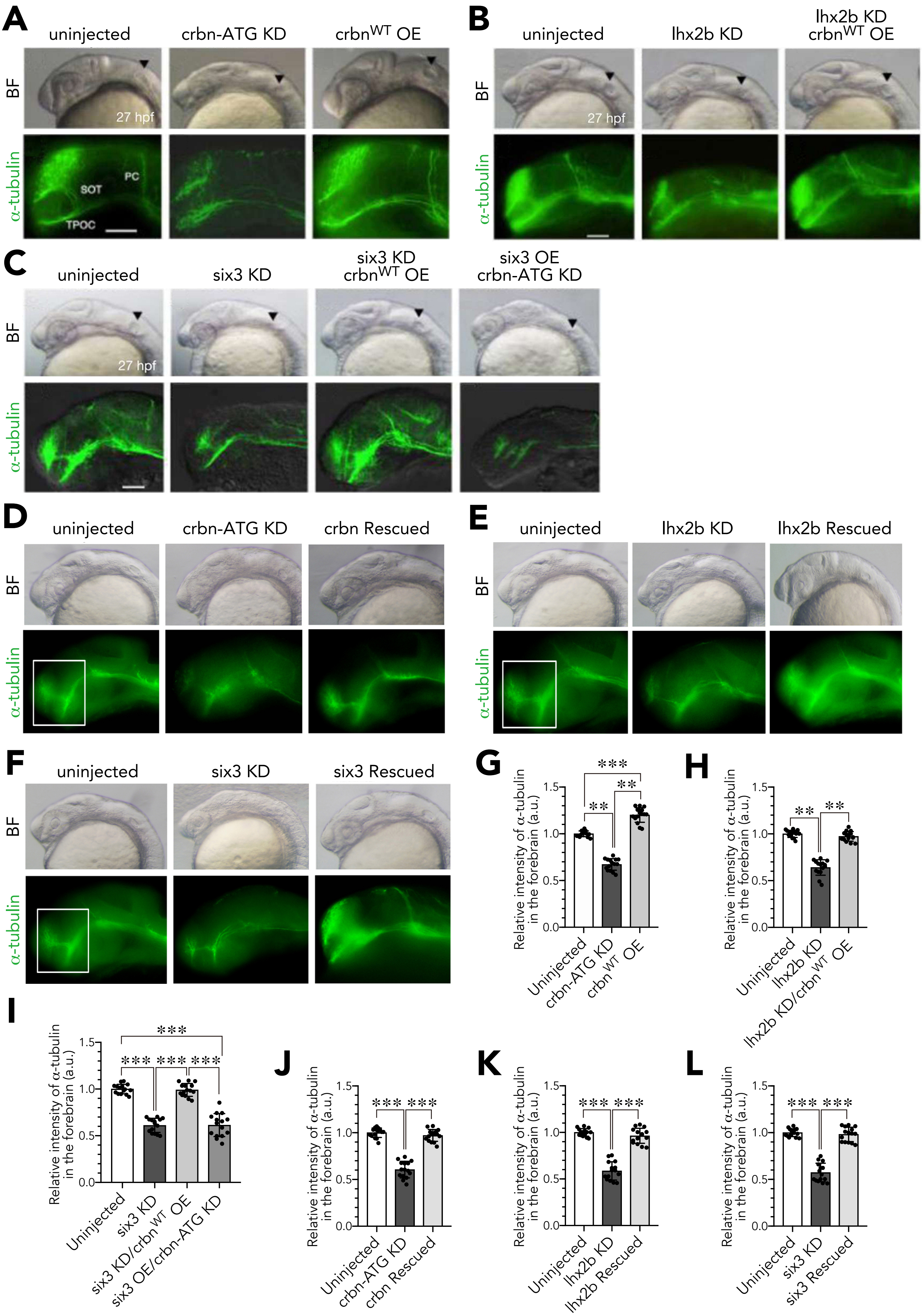

A
uninjected
crbn-ATG KD
crbnWT OE
BF
27 hpf
α-tubulin
SOT
PC
TPOC
B
uninjected
lhx2b KD
lhx2b KD
crbnWT OE
BF
27 hpf
α-tubulin
C
uninjected
six3 KD
six3 KD
crbnWT OE
six3 OE
crbn-ATG KD
BF
27 hpf
α-tubulin
D
uninjected
crbn-ATG KD
crbn Rescued
BF
α-tubulin
E
uninjected
lhx2b KD
lhx2b Rescued
BF
α-tubulin
F
uninjected
six3 KD
six3 Rescued
BF
α-tubulin
G
Relative intensity of α-tubulin in the forebrain (a.u.)
Uninjected
crbn-ATG KD
crbn WT OE
H
Relative intensity of α-tubulin in the forebrain (a.u.)
Uninjected
lhx2b KD
lhx2b KD/crbn WT OE
I
Relative intensity of α-tubulin in the forebrain (a.u.)
Uninjected
six3 KD
six3 KD/crbn WT OE
six3 OE/crbn-ATG KD
J
Relative intensity of α-tubulin in the forebrain (a.u.)
Uninjected
crbn-ATG KD
crbn Rescued
K
Relative intensity of α-tubulin in the forebrain (a.u.)
Uninjected
lhx2b KD
lhx2b Rescued
L
Relative intensity of α-tubulin in the forebrain (a.u.)
Uninjected
six3 KD
six3 Rescued

Figure S5. Suppression of *six3*- or *lhx2b*-knockdown Phenotypes by *crbn* Overexpression, Related to Figures 2 and 3.
(A–F) One-cell stage embryos were left uninjected or injected with MO and/or mRNA as indicated and then immunostained with anti-acetylated α-tubulin antibody at 27 hpf. Bright-field (upper panels) and fluorescence (lower panels) images are shown. (G–L) Fluorescence intensities of the regions indicated with rectangles in (A) to (F) were measured and normalized to the intensities of uninjected embryos and are shown as means ± SD (n = 15 per group). T, telencephalic neural cluster; PC, posterior commissure; SOT, supra-optic tract; TPOC, tract of postoptic commissure. Scale bar, 50 μm. **$P < 0.01$, ***$P < 0.001$.

Figure S6

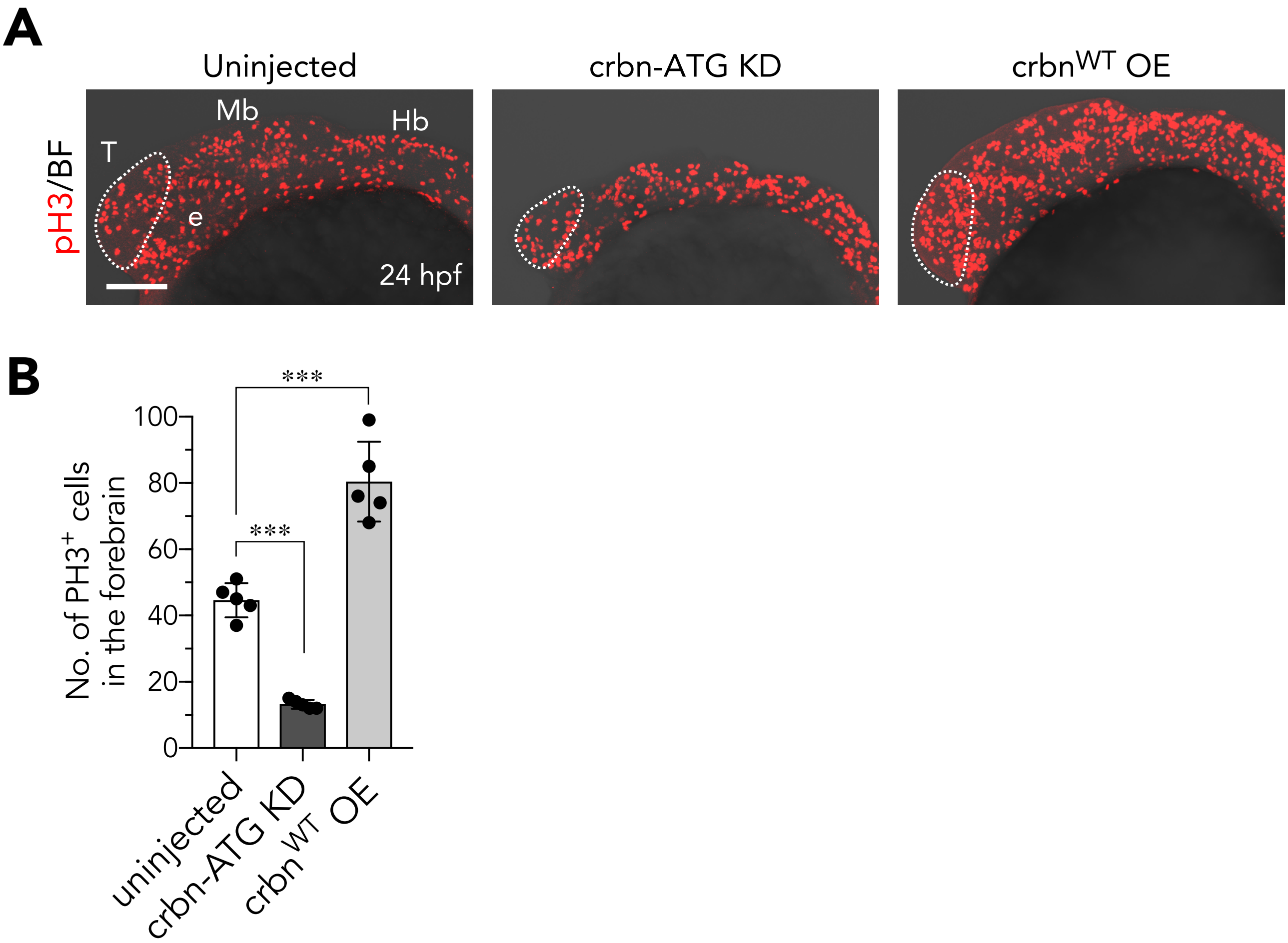
A
Uninjected
crbn-ATG KD
crbnWT OE
pH3/BF
Mb
Hb
T
e
24 hpf
B
***
***
No. of PH3+ cells in the forebrain
100
80
60
40
20
0
uninjected
crbn-ATG KD
crbn WT OE

Figure S6. Effects of *crbn* Knockdown or Overexpression on Cell Proliferation in the Brain, Related to Figures 2 and 3.

(A) Embryos left uninjected or injected with *crbn*-ATG MO or $crbn^{WT}$ mRNA were immunostained using anti-phosphorylated histone (pH3) antibody at 24 hpf to visualize proliferating cells. Fluorescence images overlaid with bright-field images are shown. e, eye; Hb, hindbrain; Mb, midbrain; T, telencephalon. Telencephalic regions analyzed in (B) are indicated with dashed lines. (B) Quantification and statistical analysis of the data shown in (A). The numbers of pH3-positive cells were counted and are shown as means ± SD (n = 5 per group). Scale bar, 100 μm. ***$P < 0.001$.

Figure S7

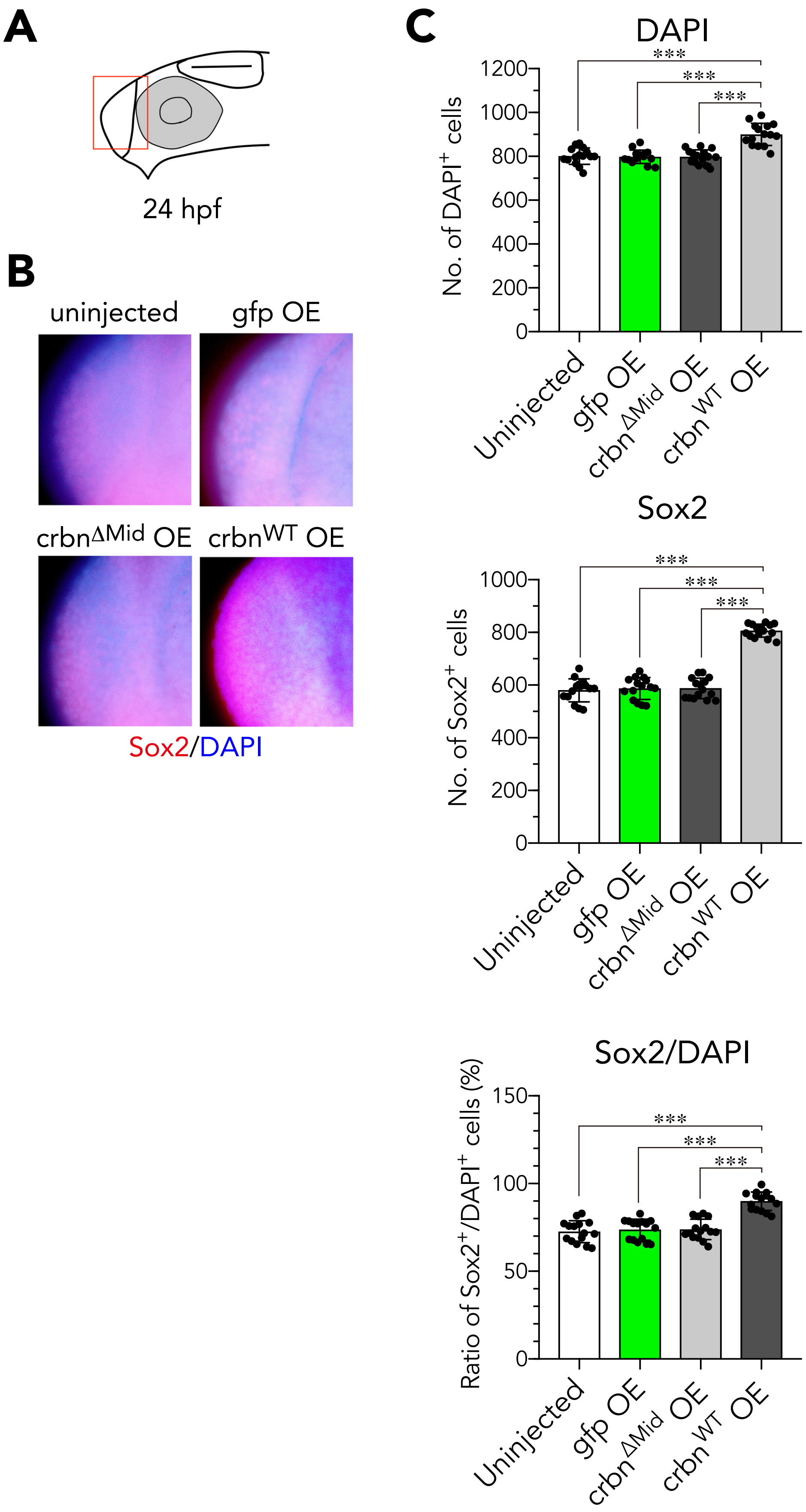

A
24 hpf
B
uninjected
gfp OE
crbnΔMid OE
crbnWT OE
Sox2/DAPI
C
DAPI
No. of DAPI+ cells
0
200
400
600
800
1000
1200
***
***
***
Uninjected
gfp OE
crbn ΔMid OE
crbn WT OE
Sox2
No. of Sox2+ cells
0
200
400
600
800
1000
***
***
***
Uninjected
gfp OE
crbn ΔMid OE
crbn WT OE
Sox2/DAPI
Ratio of Sox2+/DAPI+ cells (%)
0
50
100
150
***
***
***
Uninjected
gfp OE
crbn ΔMid OE
crbn WT OE

Figure S7. Sox2 Expression in *crbn*-overexpressing Embryos, Related to Figure 5. (A) Illustration of 24-hpf embryos. A telencephalic region analyzed in (B) and (C) are indicated with rectangle. (B) Embryos left uninjected or injected with mRNA encoding *gfp*, $crbn^{\Delta Mid}$, or $crbn^{WT}$ were immunostained using anti-Sox2 antibody and counter-stained with DAPI at 24 hpf. (C) Quantification and statistical analysis of the data shown in (B). Fluorescence images were taken as a z-series of optical sections at 50-µm intervals to accurately determine the number of DAPI-positive cells and Sox2-positive cells in a telencephalic region. Data are shown as means ± SD (n = 15 per group). ***$P < 0.001$.

Transparent Methods

Breeding of zebrafish embryos. Embryos were collected by natural matings of adult fish that were kept at 28.5°C on a 14-hours light/10-hours dark cycle. In some cases, embryos were raised in 0.003% phenylthiourea (Sigma) to inhibit pigment formation, as previously described (Elsalini *et al*., 2003). This research was approved by IACUC of Tokyo Medical University (the approval number: H290075).

Thalidomide treatment of zebrafish. Preparation of thalidomide solution and treatment of zebrafish were carried out as previously described (Ito *et al*., 2010). Briefly, thalidomide powder (Tocris Cookson) was dissolved in DMSO and added to E3 medium pre-heated at 65°C to adjust the DMSO concentration to 0.1%. It was immediately mixed by vigorous vortexing to avoid precipitation. Zebrafish embryos were dechorionated by pronase treatment for 3 min at room temperature. Dechorionated embryos were washed four times with E3 medium and then transferred to thalidomide solution and incubated at 28.5°C.

Measurement of head and optic vesicle size. The relative sizes of the head and the eye were measured at the same stage, and the ratios of head thickness and eye diameter to body axis length were calculated.

Microinjection. Antisense morpholino oligonucleotides (MO) and/or capped RNAs were microinjected into 1-cell stage embryos by using a nitrogen gas-pressure microinjector IM 300 (Narishige) as previously described (Ito *et al*., 2010). Conditions for microinjection were as follows: gas pressure, 15 picosiemens; release period, 15 to 45 milliseconds per single injection. The optimal doses of MOs were determined as follows: MO solutions were prepared at 2 ng/nl. Prior to each experiment, injection volume was estimated by measuring the diameter of a droplet injected into mineral oil by using an eyepiece micrometer and was calibrated so that approximately 1 nl was injected per puff. One nanogram of MOs were used to knock down *crbn*, whereas 2 ng of MOs were used to knock down *cul4a* and *cul4b*. Capped RNAs were synthesized by using the mMESSAGE mMACHINE in vitro transcription kit (Ambion) and cDNAs cloned into pCS2^{+} (Turner and Weintraub, 1994). Unless stated otherwise, 300 pg of capped mRNAs were used for rescue or overexpression.

The sequences of MOs (Gene Tools) used in this study are as follows: *crbn*-ATG KD (translation-blocking), 5′-GCTGGTTCCCCATTTCGTCGTTAAT-3′; *crbn*-ATG 5-mis, 5′-GCAGCTTCCGCATTTCCTCCTTAAT-3′; *crbn*-Spl KD (splice-blocking), 5′-CTGTGGAAAAACAGAAACGCAGATA -3′; *crbn*-Spl 5-mis, 5′-CTGTGCAAAAAGACAAACCCACATA -3′; *cul4a* KD (translation-blocking), 5′-CTGGTGCTGAACATCTTCTGCCATC-3′; *cul4a* 5-mis, 5′ -CTCGTGCTCAACATCTTGTCCGATC-3′; *cul4b* KD (translation-blocking), 5′-TCGCTAATCTACTAATGCTACGATG-3′; *cul4b* 5-mis, 5′-TCCCTAATGTAGTAATCCTACCATG-3′; *six3* KD (translation-blocking), 5′-GCTCTAAAGGAGACCTGAAAACCAT-3′; *lhx2b* KD (translation-blocking), 5′-TCTGCAACCCAAGATTTCCGTGAGA-3′; *p53* KD (translation-blocking) 5′-GCGCCATTGCTTTGCAAGAATTG-3′. The *crbn* MOs used in this study are different from what we used in Ito *et al.* (2010) and were newly designed for this study. A number

of experiments were conducted to check the specificity of these MOs. The *cul4a* MO used in this study was first reported in Ito *et al.* (2010), in which knockdown phenotypes caused by this MO were more thoroughly studied and described. The *cul4b* MO used in this study was derived from Zhao *et al.* (2015), in which the authors performed phenotypic analysis of *cul4a*- or *cul4b*-knockdown embryos and observed defects in pectoral fin development, in agreement with our previous finding (Ito *et al.*, 2010). The *lhx2b* MO used in this study was first reported in Ando *et al.* (2005). In this and subsequent studies (Ando *et al.*, 2005; Ando and Okamoto, 2006), knockdown phenotypes caused by this MO were more thoroughly studied and described. The *six3* MO used in this study was first reported in Ando *et al.* (2005). In zebrafish, there are three *six3*-related genes, *six3a*, *six3b*, and *six7*. Although this MO was originally designed for *six3b*, the MO inhibits the expression of not only *six3b* but also *six3a* due to a high sequence similarity and has been used as a dual-specificity MO in many studies (McCollum *et al.*, 2007; Sanek *et al.*, 2009; Lenkowski *et al.*, 2013; Bhatia *et al.*, 2015). The *p53* MO used in this study was derived from Langheinrich *et al.* (2002). This MO has been used in over 600 papers and is a common reagent for apoptosis research in zebrafish.

Whole-mount *in situ* hybridization. Whole-mount *in situ* hybridization was carried out as previously described (Thisse and Thisse, 2008).

Reverse transcription PCR. Total RNAs were isolated from embryos by using Sepasol-RNA I Super G (Nacalai Tesque) and further purified by using RNeasy Plus Mini Kit (Qiagen). Reverse transcription was performed by using ReverTra Ace qPCR RT Kit (Toyobo) and a mixture of random hexamer and oligo-dT primers. PCR was carried out using KOD Plus Neo (Toyobo).

Immunohistochemistry. Immunohistochemistry was carried out by incubating embryos with primary antibodies against acetylated α-tubulin (Sigma, T7451), GFAP (ZIRC, zrf-1), serotonin (Abcam, ab66047), and Sox2 (GeneTex, GTX124477), followed by incubation with secondary antibodies against rabbit or mouse IgG conjugated with Alexa Fluor (Molecular Probes). Samples were mounted on slide glasses, and images were obtained using a fluorescence microscope. Labeling of proliferating cells in the forebrain was carried out as previously described (Ando *et al.*, 2005). Proliferating or apoptotic cells were immunostained with anti-phosphorylated histone H3 (pH3) antibody (Sigma, H0412) or anti-active Caspase-3 antibody (BD, 559565), respectively. For Sox2 labeling, embryos labeled with Alexa Fluor 594-conjugated anti-rabbit IgG (Molecular Probes) and DAPI were observed laterally at low magnification (x100), and then the number of cells labeled with Sox2 and DAPI were counted using a z-series of confocal images at 50-μm intervals at high magnification (x400). The focal plane was adjusted for each embryo using the presumptive forebrain ventricle, a narrow space between ventral telencephalon and diencephalon, as a guide (Lowery and Sive, 2005).

Statistical analysis. GraphPad Prism was used for unpaired t test with Welch's correction, Kruskal-Wallis test with Dunn's post hoc test and one-way ANOVA with Bonferroni's post hoc test. Probability values (P values) < 0.05 were considered to be statistically significant (*$P < 0.05$, **$P < 0.01$, ***$P < 0.001$). Values indicated are means ± SD.

Supplemental References

Ando, H., Kobayashi, M., Tsubokawa, T., Uyemura, K., Furuta, T., and Okamoto, H. (2005). Lhx2 mediates the activity of Six3 in zebrafish forebrain growth. Dev. Biol. 287, 456-468.

Ando, H., and Okamoto, H. (2006). Efficient transfection strategy for the spatiotemporal control of gene expression in zebrafish. Mar. Biotechnol. 8, 295-303.

Bhatia, S., Gordon, C.T., Foster, R.G., Melin, L., Abadie, V., Baujat, G., Vazquez, M.P., Amiel, J., Lyonnet, S., van Heyningen, V., *et al.* (2015). Functional assessment of disease-associated regulatory variants in vivo using a versatile dual colour transgenesis strategy in zebrafish. PLoS Genet. 11, e1005193.

Elsalini, O.A., and Rohr, K.B. (2003). Phenylthiourea disrupts thyroid function in developing zebrafish. Dev. Genes Evol. 212, 593-598.

Ito, T., Ando, H., Suzuki, T., Ogura, T., Hotta, K., Imamura, Y., Yamaguchi, Y., and Handa, H. (2010). Identification of a primary target of thalidomide teratogenicity. Science 327, 1345-1350.

Langheinrich, U., Hennen, E., Stott, G., and Vacun, G. (2002). Zebrafish as a model organism for the identification and characterization of drugs and genes affecting p53 signaling. Curr. Biol. 12, 2023-2028.

Lenkowski, J.R., Qin, Z., Sifuentes, C.J., Thummel, R., Soto, C.M., Moens, C.B., and Raymond, P.A. (2013). Retinal regeneration in adult zebrafish requires regulation of TGFβ signaling. Glia 61, 1687-1697.

Lowery, LA., and Sive, H. (2005) Initial formation of zebrafish brain ventricles occurs independently of circulation and requires the nagie oko and snakehead/atp1a1a.1 gene products. Development 132, 2057-2067.

McCollum, C.W., Amin, S.R., Pauerstein, P., and Lane, M.E. (2007). A zebrafish LMO4 ortholog limits the size of the forebrain and eyes through negative regulation of six3b and rx3. Dev. Biol. 309, 373-385.

Sanek, N.A., Taylor, A.A., Nyholm, M.K., and Grinblat, Y. (2009). Zebrafish zic2a patterns the forebrain through modulation of Hedgehog-activated gene expression. Development 136, 3791-3800.

Thisse, C., and Thisse, B. (2008) High-resolution in situ hybridization to whole-mount zebrafish embryos. Nat. Protoc. 3, 59-69.

Turner, DL., and Weintraub, H. (1994) Expression of achaete-scute homolog 3 in Xenopus embryos converts ectodermal cells to a neural fate. Genes Dev. 8, 1434-1447.

Zhao, X., Jiang, B., Hu, H., Mao, F., Mi, J., Li, Z., Liu, Q., Shao, C., and Gong, Y. (2015). Zebrafish cul4a, but not cul4b, modulates cardiac and forelimb development by upregulating tbx5a expression. Hum. Mol. Genet. 24, 853-864.
